# Biodegradable Liquid Slow-Release Mulch Film Based on Bamboo Residue for Selenium-Enriched Crop Cultivation

**DOI:** 10.34133/research.0685

**Published:** 2025-05-12

**Authors:** Chaoqi Chen, Zhaoshuang Li, Kuaile Yin, Lei Li, Zhen Zhang, Xu Xu, He Liu, Yan Qing, Xingong Li, Yiqiang Wu

**Affiliations:** ^1^ College of Materials Science and Engineering, Central South University of Forestry & Technology, Changsha 410004, China.; ^2^ International Innovation Center for Forest Chemicals and Materials, Nanjing Forestry University, Nanjing 210037, China.; ^3^ Key Laboratory of Biomass Energy and Material, Institute of Chemical Industry of Forestry Products, Chinese Academy of Forestry, Nanjing 210042, Jiangsu Province, China.

## Abstract

The development of biodegradable mulch film is an effective means to address plastic pollution and promote modern green agriculture. In this work, with compounding sodium carboxymethyl cellulose (CMC) and quaternized lignin (QL), a biodegradable liquid mulch film (PVA@CMC/QL) was constructed by introducing polyvinyl alcohol (PVA) and a selenium-containing cross-linking agent through electrostatic interaction. The effect of sodium carboxymethyl cellulose and QL on different liquid mulch films was examined. PVA@CMC/QL had exceptional spray-film-forming properties of liquid mulch film and was capable of generating a dense mulch film above the soil/on top of the soil under natural conditions. PVA@CMC/QL exhibited excellent oxygen transmission rate (60.2 cm^3^·m^−2^·d^−1^·Pa^−1^) and water vapor transmission rate (753.4 g·m^−2^·d^−1^). Soil temperature and humidity increased by 0.4 to 2.1 °C and 0.5% to 2.8%, respectively, in the soil covered with PVA@CMC/QL compared to those in other controls, thereby confirming its exceptional moisture retention and insulation capabilities. PVA@CMC/QL combined remarkable weed suppression with only 13.3% weed germination under the mulch. Optimal rhizome growth of pak choi seedlings was observed under the PVA@CMC/QL cover, as demonstrated by the planting of both pak choi seedlings and weeds. Roots and stems increased by 3.8 ± 0.3 and 1.2 ± 0.3 cm, respectively. The weed suppression mechanism of PVA@CMC/QL was explained through the lens of density functional theory. In addition, the selenium content of pak choi seedlings under PVA@CMC/QL cover could reach 28.5 μg/kg, making the mulch film both degradable and highly reusable. This work not only improved the value-added utilization of bamboo residues but also gave new insight into the research on multifunctional bamboo–plastic mulch film.

## Introduction

Mulch film has become increasingly valued in contemporary agriculture due to its numerous benefits, including enhancing soil moisture retention, regulating soil temperature, fostering beneficial microbial activity, and controlling weed growth, all of which contribute to improving crop output and quality [1,2]. However, plastic mulch film is generally prepared from polyethylene (PE) or other petrochemical materials, most of which degrade slowly or nondegradably [3,4]. Residual plastic in the soil covered by long-term mulch film accumulates over time, causing “white pollution” on farmland [5,6]. Mulch film residues tend to impede soil water infiltration, affect soil hygroscopicity, and reduce soil permeability, microbial activity, and soil fertility levels, ultimately affecting soil quality and crop yields [7,8]. Therefore, the development of biodegradable mulch film is essential to safeguarding agricultural practices [9]. Liquid mulch film with an organic polymer as the main carbon skeleton is available as a new type of fully biodegradable agricultural mulch film, which is able to be applied to the soil surface via machine spraying [10–12]. At the time that the liquid mulch film evaporates on the soil surface, it forms a polymer mesh membrane that closes the soil surface pores, thereby limiting moisture evaporation without affecting water infiltration while also providing heat preservation and moisture retention [13,14]. Meanwhile, the liquid mulch film has strong adhesion to soil, which bonds and disperses soil particles to form a continuous granular structure that enhances soil structure, stabilizes the topsoil, and protects the till layer [15,16]. However, most of the early products of liquid mulch film are prepared by modification of paper black liquor with petroleum asphalt, lignite, or peat [17]. Petroleum asphalt is nonrenewable, and asphalt contains anthracene, phenanthrene, and pyridine, which are toxic substances that pollute the soil during the degradation process [18]. Therefore, it is crucial to seek green and biodegradable biomass raw materials for the preparation of liquid mulch film.

With a global cultivation area of over 36 million hectares and abundant production, bamboo is an important biomass material [19–21]. Known for being fast growing, renewable, biodegradable, and environmentally friendly, bamboo is considered a promising alternative to plastic [22–24]. Furthermore, the utilization of bamboo processing in China, including the variety of products, scale, and output, is among the foremost in the world [25,26]. Nevertheless, a large amount of solid residues, including bamboo shavings, bamboo foam, and bamboo tips, contains approximately 43% to 47% cellulose and is consistently produced during the processing of bamboo [27,28]. Conventional treatment is incineration or charcoal production, which fails to efficiently utilize the residue waste of resources and leads to environmental pollution [29,30]. Therefore, there is an urgent need for a technology or means to efficiently utilize bamboo residues.

Bamboo cellulose is a valuably renewable resource with fast growth, high mechanical strength, heat and corrosion resistance, biocompatibility, and biodegradability. It is frequently transformed into high-performance composites, bioplastics, and nanomaterials [31–35]. The liquid mulch film, prepared from synthetic polymer compounds of bamboo cellulose through the introduction of functional modifiers, overcomes the shortcomings of the third-generation liquid mulch film (crop straw or modified peat as raw material) with poor film-forming properties and a single function [36,37]. It is undeniable that the use of bamboo residue, converted into biodegradable liquid mulch film to replace traditional plastic mulch film, plays a key role in addressing plastic pollution and increasing the value-added potential of bamboo.

Currently, most biodegradable mulch films degrade directly into the soil, resulting in a waste of resources. Therefore, it is critical to realize the waste reuse of degraded mulch film [38]. Bio-based, biodegradable liquid mulch film can be applied in agricultural production to create slow-release substrates for pesticides, fertilizers, and plant growth regulators [39]. The active ingredients are released slowly at an appropriate rate, which prolongs the duration of action. This process facilitates crop uptake, reduces wastage and usage, and prevents the overapplication of pesticides and fertilizers [40]. At present, liquid mulch films with selenium-containing slow-release features have been little researched. Selenium is an essential micronutrient, and its deficiency may cause serious health problems [41]. It is regrettable that approximately 1 billion people worldwide are still selenium deficient [42]. The use of selenium fertilizers has been shown to be a viable way to increase the selenium content of food and to address selenium deficiency in humans [43,44]. The combination of mulch film degradation and slow release of selenium fertilizer is achieved by introducing selenium-containing compounds into the polymer chain, directly converting the mulch film into value-added selenium fertilizer [45]. Using selenium fertilizers applied via liquid mulch film can notably enhance plant selenium absorption, providing an effective approach to mitigating the effects of selenium deficiency globally.

**Fig. 1.**
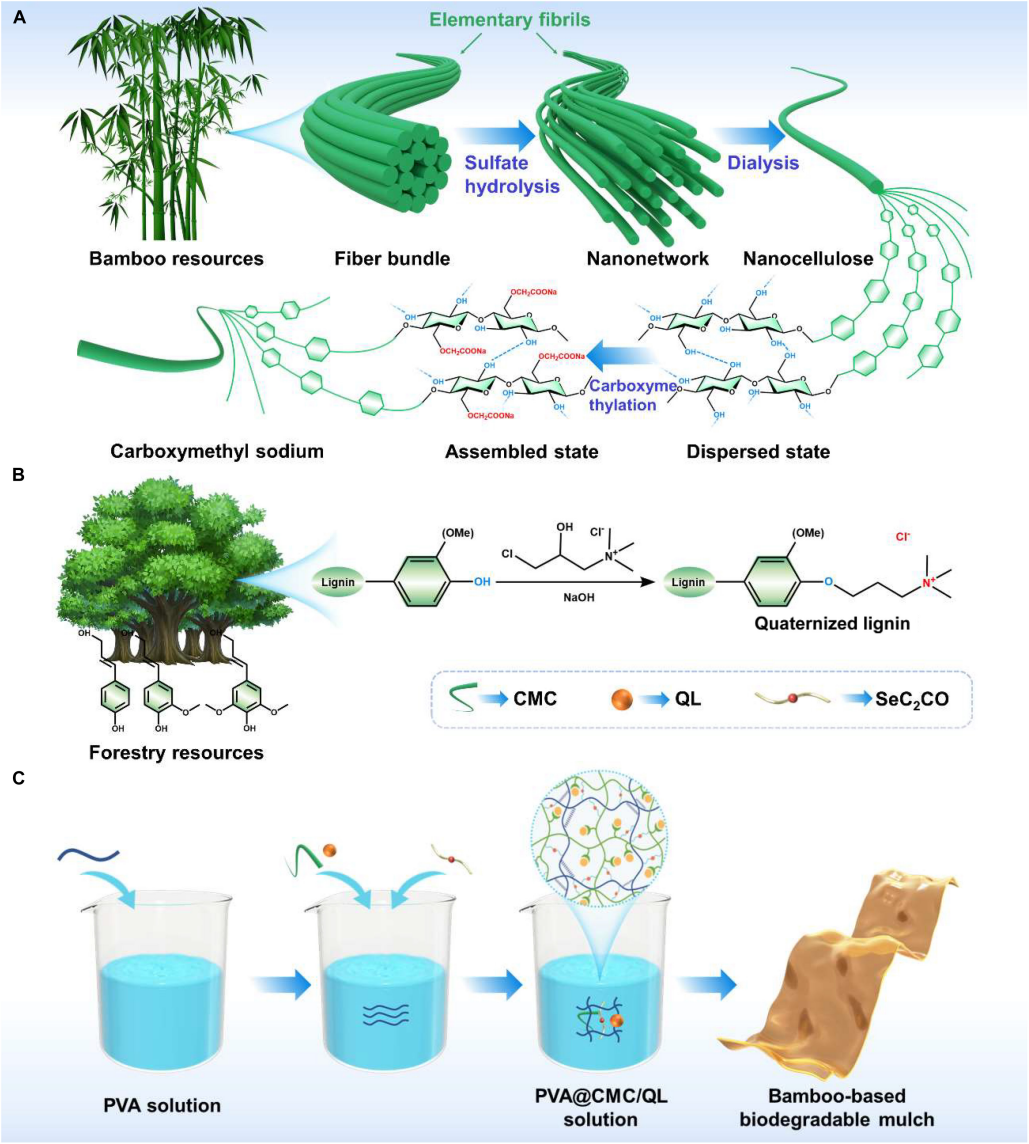
(A) Preparation of sodium carboxymethyl cellulose from bamboo residues. (B) Synthesis of quaternized lignin via quaternization modification. (C) Fabrication of PVA@CMC/QL-based liquid mulch film. PVA, polyvinyl alcohol; CMC, sodium carboxymethyl cellulose; QL, quaternized lignin.

In this work, the electrostatic interaction between sodium carboxymethyl cellulose (CMC) and quaternized lignin (QL) was employed to introduce polyvinyl alcohol (PVA) and cross-linking by selenium-containing diol compounds (SeC_2_CO) (Fig. 1A to C). This approach led to the development of a multifunctional bamboo-based liquid mulch film (PVA@CMC/QL) that combined the properties of moisture and heat preservation, weed suppression, and slow release of selenium fertilizer. Experimental tests, including those on tensile strength, elongation at break, water resistance, light transmission, air permeability, thermal retention, and moisture conservation for the soil, were carried out on PVA@CMC/QL. The pot test showed that PVA@CMC/QL promoted the growth of pak choi seedlings and inhibited weeds. Meanwhile, the excellent soil degradation performance of PVA@CMC/QL was revealed, with a degradation rate as high as 83.5% in about 45 d. Simultaneously with the degradation of PVA@CMC/QL soil, the selenium-containing compounds oxidatively decomposed to selenate and monomeric selenium, providing a slow-release effect of selenium fertilizer. In addition, a full life cycle assessment (LCA) was conducted to compare the ecological effects of PVA@CMC/QL with those of commercially available mulch films, both nonbiodegradable and biodegradable. The preparation of bamboo-based multifunctional liquid slow-release mulch film not only expands the direction of bamboo utilization but also fulfills the “bamboo instead of plastic” initiative and realizes the mission of the “dual-carbon” strategy of the times.

## Results and Discussion

### Properties of PVA@CMC/QL

**Fig. 2.**
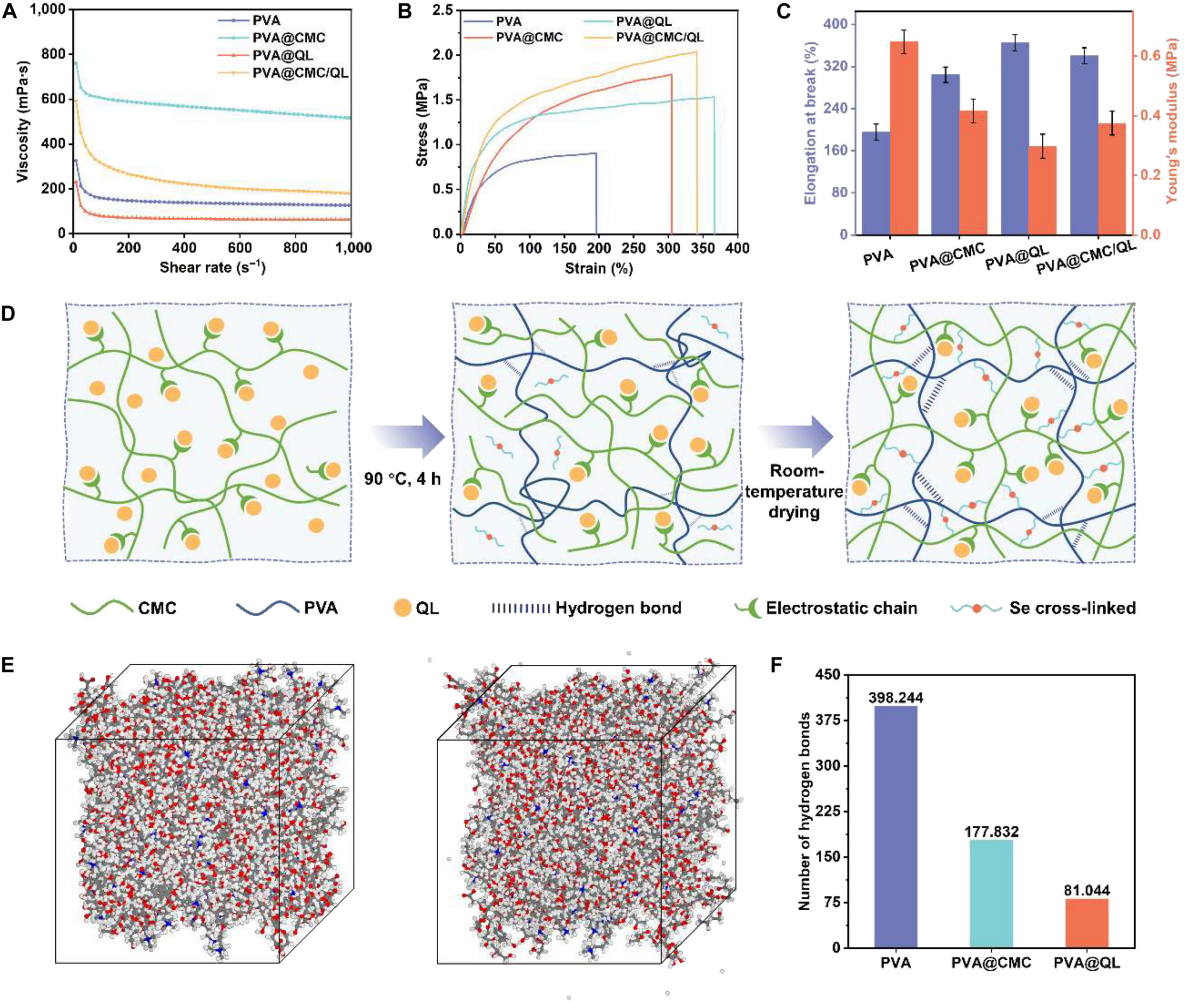
(A) Viscosity test of different samples at different shear rates. (B) Tensile stress–strain curves and (C) elongation at break and Young’s modulus of different samples. (D) Cross-linking schematic and (E) molecular dynamics simulations of PVA@CMC/QL. (F) Number of hydrogen bonds for PVA, PVA@CMC, and PVA@QL.

In practice, mulch film with high tensile strength is better able to resist external forces, reducing the risk of rupture and prolonging service life [46]. The 3-dimensional response surface plots in Fig. S2 demonstrate the joint influence of CMC and QL on tensile strength, as analyzed using a central composite design model with 2 factors and 3 levels. The tensile strength of PVA@CMC/QL improved gradually with increasing CMC and QL content. The best tensile strength (2.1 MPa) was obtained when the CMC-to-QL mass fraction ratio was 5:15. When the mass percentage was greater than 5:15, the tensile strength did not continue to increase but tended to decrease. Therefore, the optimal ratio of CMC and QL for the preparation of PVA@CMC/QL was determined through modeling. Liquid viscosity was the main factor influencing the formation of the spray shape. A lower viscosity facilitated the liquid’s passage through the spray gun, promoting uniform coverage of the soil surface and the formation of a complete mulch film. As shown in Fig. 2A, the viscosity of the 4 samples decreased with increasing shear rate, which was a pseudoplastic fluid behavior. When the viscosities of PVA, PVA@QL, and PVA@CMC/QL were controlled in the range of 236 to 598 mPa·s, these liquid mulch films could be sprayed on top of the soil with a sprayer. PVA@CMC (786 mPa·s) was more viscous, and the fluid molecules in the sample encountered greater resistance when flowed through the fine gun nozzle. As a result, PVA@CMC was difficult to spray uniformly into a mist to cover the soil surface, making it unsuitable for spraying. The permeability of a liquid mulch film was the depth of penetration of the liquid on top of the soil through the capillary action of the soil into the soil layer. As the permeability decreased, the liquid remained on the soil surface for a longer time, which facilitated the formation of a film. As illustrated in Fig. S3, the soil penetration depths of PVA, PVA@CMC, PVA@QL, and PVA@CMC/QL were 0.8, 0.5, 0.7, and 0.6 cm, respectively; although PVA@CMC had the lowest penetration, its high viscosity hindered its ability to be seamlessly sprayed. In contrast, PVA had the highest permeability, which resulted in its inability to stay in the soil for a long time to form a mulch film. Neither was suitable for the preparation of sprayable liquid coverings. PVA@CMC/QL, however, formed a homogeneous “soil film” upon the soil surface. A stable and uniform membrane layer was distinctly visible on the outer layer of the liquid membranes, which could be ascribed to the strengthened hydrogen bonding interactions between the PVA@CMC/QL components, leading to a thick and flat interface (Fig. S4). Although PVA@QL was sprayable on the soil surface, the mulch film formation was discontinuous and incomplete, probably due to insufficient intermolecular forces in the liquid state. Furthermore, PVA@CMC/QL was able to form a continuous and intact film on the soil surface and adhere some of the soil particles below the soil surface to form a layer of clods (Fig. S5). Under the same conditions, PVA failed to form stable soil blocks. The integrity and thickness of the soil blocks formed by PVA@CMC and PVA@QL were both inferior to those formed by PVA@CMC/QL.

Mulch film with a high elongation at break reduces compression on the crop root system in a taut state and promotes crop growth. Therefore, liquid mulch film with excellent mechanical properties ought to have good tensile strength and elongation at break. As indicated by the stress–strain curves of the liquid mulch films (Fig. 2B), the tensile strength of PVA@CMC/QL was substantially greater than those of the other liquid mulch films, indicating that the ionic bonding cross-linking of QL and CMC boosted the mechanical characteristics of mulch films. The measurements of Young’s modulus and elongation at break revealed that PVA@QL and PVA@CMC/QL exhibited increased elongation at break and decreased Young’s modulus (Fig. 2C). Specifically, the elongation at break of PVA, PVA@CMC, PVA@QL, and PVA@CMC/QL was 195%, 306%, 358%, and 340%, respectively, indicating that QL improved the toughness of the liquid mulch film. Although the elongation at break of PVA@QL slightly outperformed that of PVA@CMC/QL, an excessively high elongation at break could lead to easy deformation of the mulch film when subjected to force, potentially compromising its stability and durability. With regard to combined tensile strength and elongation at break, the mechanical properties of PVA@CMC/QL performed well. In addition, the cross-linking mechanism of PVA@CMC/QL is presented in Fig. 2D. CMC and QL were held together by electrostatic interactions, whereas SeC_2_CO was cross-linked with PVA through the CMC/QL complex. As a result, PVA@CMC/QL, which was cross-linked by electrostatic interactions, selenium-containing cross-linkers, and hydrogen-bonded multivariate cross-linking, displayed excellent mechanical properties. In addition, the free diffusion of PVA@CMC/QL molecules within 50 ps was simulated by molecular dynamics (Fig. 2E). The number of hydrogen bonds in PVA, PVA@CMC, and PVA@QL are compared in Fig. 2F. The introduction of CMC and QL led to a marked reduction in the quantity of hydrogen bonds within PVA, suggesting that CMC and QL interfered with the hydrogen bond construction in PVA to different extents and remodeled the formation of the cross-linked network [47].

### Microstructures and chemical components

**Fig. 3.**
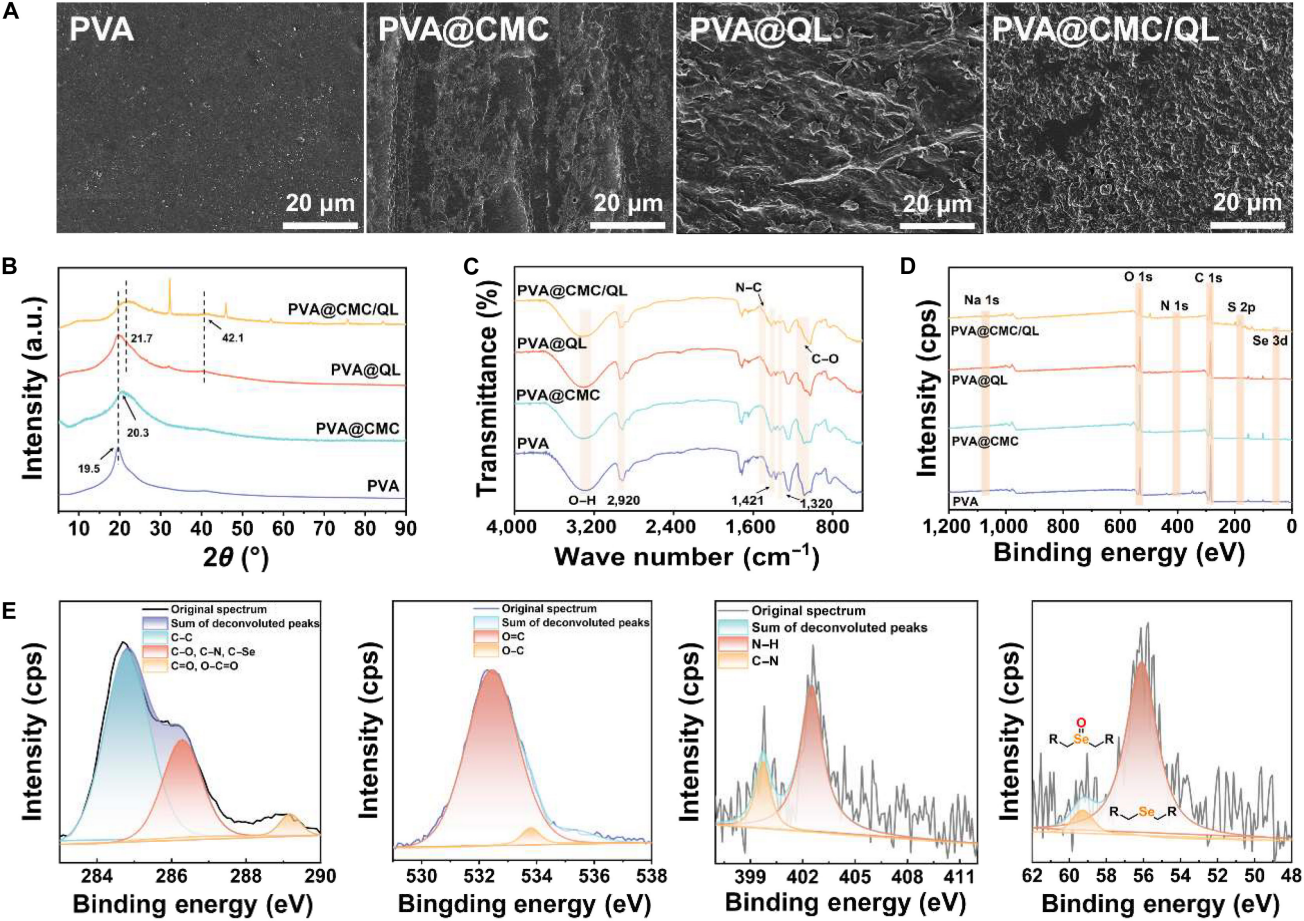
Features, morphology, and chemical composition of the samples. (A) Scanning electron microscopy (SEM) images characterizing liquid mulch films. (B) X-ray diffraction (XRD) curves and (C) Fourier transform infrared (FT-IR) spectra of liquid mulch films. (D) X-ray photoelectron spectroscopy (XPS) broad-scan spectra of different samples and (E) high-resolution XPS spectra of PVA@CMC/QL.

The microstructure of PVA@CMC/QL was analyzed by scanning electron microscopy characterization, which facilitated the comprehension of the morphological changes in the liquid mulch film by the introduction of CMC and QL. As shown in Fig. 3A, the originally flat PVA surface showed a different rough morphology with the introduction of CMC and QL, respectively. An irregular morphology was presented on both the PVA@CMC and PVA@QL surfaces. CMC and QL were cross-linked by electrostatic interactions, and aggregation of CMC/QL was clearly observed on the PVA@CMC/QL surface. Meanwhile, the homogeneous dispersion of each element upon the exterior of PVA@CMC/QL was observed by an energy-dispersive spectrometer (Fig. S5). It was observed that the diffraction peak of PVA was at 2*θ* = 19.5°, and with the introduction of CMC, a strong diffraction peak appeared near 2*θ* = 20.3°, as seen in Fig. 3B. The decrease in crystallinity was primarily attributed to hydrogen bonds generated among the –OH groups of PVA and CMC, which hindered the dynamics of PVA molecular chains. The crystallization peaks of PVA@QL occurred at 2*θ* = 21.7° and 42.1°. QL resulted in an enlargement of the agglomerate particle size, which in turn gradually reduced the crystallinity of the mulch film. In addition, the characteristic absorption peak of the C–N bond in –NH^+^_4_ appeared near 1,389 cm^−1^ for PVA@CMC/QL, as shown in Fig. 3C, confirming that the quaternary ammonium group had been successfully introduced into the lignin structure. CMC exhibited an infrared characteristic peak of O–H near 3,425 cm^−1^ and infrared characteristic peaks of CH_2_ near 2,920, 1,421, and 1,320 cm^−1^, which corresponded to the asymmetric telescopic vibration, bending vibration, and out-of-plane rocking vibration of CH_2_ in CMC, respectively.

In Fig. 3D, the emergent Se 3d peak newly appeared at 55.7 eV in PVA@CMC/QL. New Na 1s peaks at 1,071.2 eV were observed in PVA@CMC/QL and PVA@CMC, while PVA@QL displayed new N 1s and S 2p peaks at 168.1 and 55.7 eV, respectively. The deconvolution x-ray photoelectron spectroscopy (XPS) spectra of C 1s exhibited 3 main peaks (Fig. 3E). The binding energy peak of PVA@CMC/QL at 284.7 eV corresponds to C–C. The peak at 286.1 eV indicated the presence of C–O, C–Se, and C–N, and the peak at 289.1 eV should be attributed to C=O and O–C=O. The high-resolution O 1s spectrum had 2 fitted peaks, representing the C–O and C=O groups, respectively, which confirmed carboxyl groups existing on the surface of PVA@CMC/QL. C–N and N–H in the high-resolution spectra of N 1s were the characteristic peaks of elemental N in the quaternary ammonium group (–NH^+^_4_), proving the successful introduction of –NH^+^_4_ into the lignin structure. Selenium XPS examination revealed the presence of a selenide structure in PVA@CMC/QL. Amorphous selenium has polymer chains generated by Se–Se bonds, and its inherent features gave it crystalline behavior, comparable to that of organic polymers. In summary, the successful construction of PVA@CMC/QL was confirmed by microstructure and chemical composition characterization.

### Moisturizing and insulating applications of PVA@CMC/QL

**Fig. 4.**
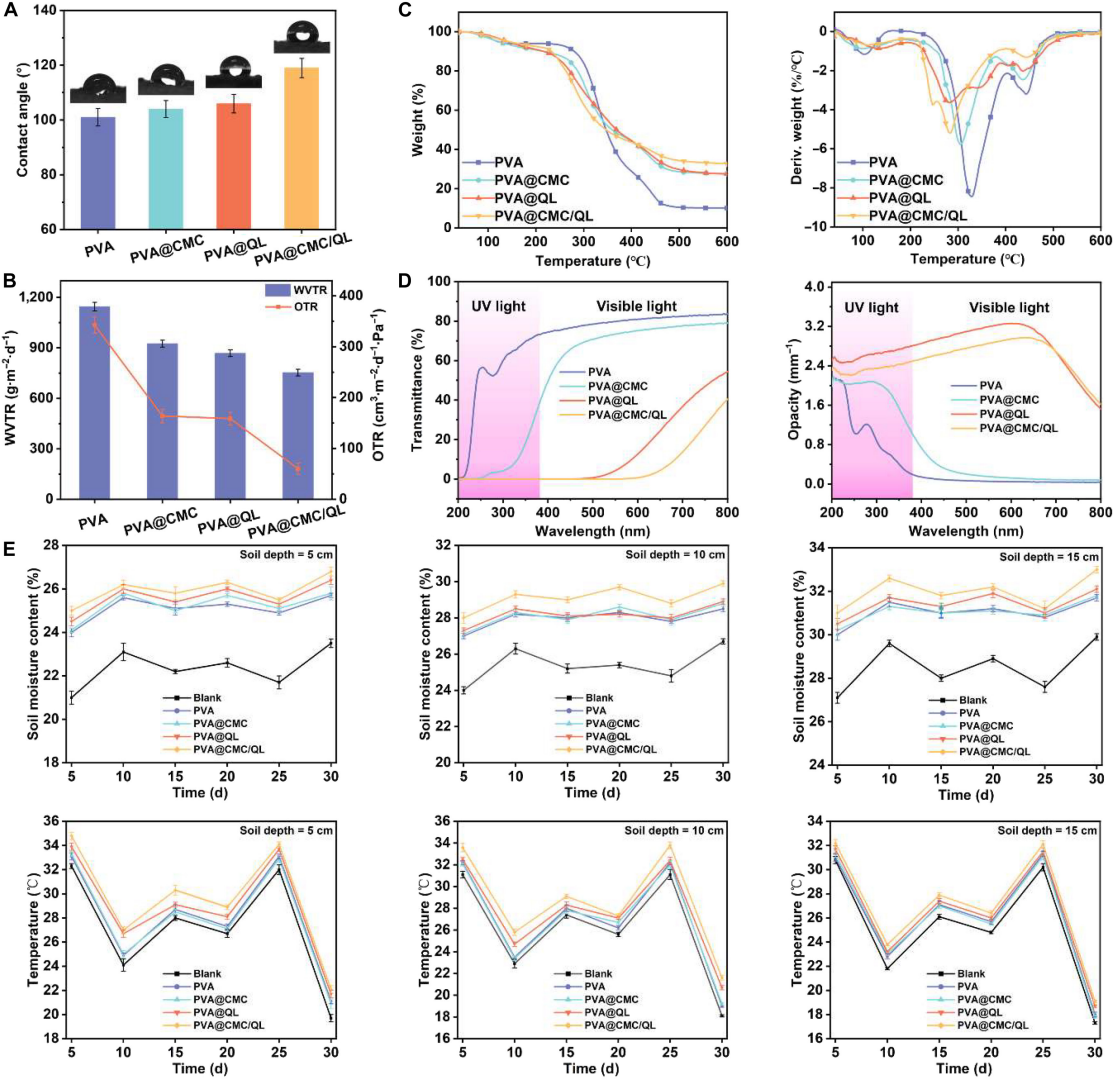
(A) Contact angle on the soil surface after liquid mulch film cover. (B) Oxygen permeability, (C) thermogravimetric analysis, and (D) ultraviolet–visible (UV–vis) transmittance spectra and opacity of liquid mulch films. (E) Effect of PVA, PVA@CMC, PVA@QL, PVA@CMC/QL, and blank control groups on soil moisture and soil temperature at different depths. WVTR, water vapor transmission rate; OTR, oxygen transmission rate.

The contact angle characterizes the ability of a liquid mulch film to adhere to soil. A larger contact angle illustrates a lower permeability and a greater tendency for the liquid mulch film to form a film on the soil surface. The contact angle of PVA@CMC/QL after film formation on soil was as high as 119.5°, which was obviously higher than those of PVA (101.0°), PVA@CMC (104.2°), and PVA@QL (106.1°) (Fig. 4A). It was attributed to the uniform film formation that allowed water droplets to stay better on the top soil layer. Mulch films with low oxygen transmission rate and water vapor transmission rate can effectively prevent soil moisture evaporation and oxygen loss, maintain soil temperature, and create a more stable growth environment for crop roots [48]. As shown in Fig. 4B, the water vapor transmission rate of PVA@CMC/QL was only 753.4 g·m^−2^·d^−1^, which was much lower than that of PVA at 1,145.1 g·m^−2^·d^−1^. This indicated that PVA@CMC/QL had excellent water vapor barrier properties. At the same time, PVA@CMC/QL demonstrated excellent oxygen barrier performance with an oxygen transmission rate of only 60.2 cm^3^·m^−2^·d^−1^·Pa^−1^. Therefore, compared to other samples, PVA@CMC/QL was able to retain more oxygen and moisture in the soil, providing favorable conditions for crop growth.

The thermal stability of the mulch film was assessed through thermogravimetry analysis and derivative thermogravimetry, which allowed for the evaluation of mass loss and structural changes during degradation, providing insight into its degradation performance. As shown in Fig. 4C and Table S1, the initial decomposition of PVA occurred at 293.2 °C, showcasing a peak weight loss rate of 8.44%/min, and leaving behind a residual mass of merely 10.15%. The initial decomposition of PVA@CMC and PVA@QL advanced from 293.2 to 276.8 and 248.3 °C with the incorporation of QL and CMC into PVA, respectively. The original decomposition of PVA@CMC/QL occurred at 232.5 °C with weight loss to 247.1 °C and then peaked at 282.9 °C. This decomposition process was attributed to pyrolysis of CMC and QL, which exhibited a peak weight loss rate of 5.21% per minute. After tests, 32.78% residue remained. This confirmed the good thermal stability of PVA@CMC/QL.

In general, ultraviolet (UV) light from the sun tends to deactivate photosensitive herbicides, insecticides, and organic fertilizers in the soil. Consequently, there was a requirement to incorporate additives capable of absorbing UV rays to conventional plastic mulch film in order to enhance the UV-shielding properties of mulch film [49]. In Fig. 4D, the transmittance of PVA and PVA@CMC reached 73.2% and 37.6% in the 200- to 380-nm UV region, respectively. In contrast, PVA@CMC/QL and PVA@QL, which contained QL, had almost zero transmittance in the UV region. This was attributed to the favorable UV absorption of lignin containing quinones, chalcones, and catalytic functional groups, which substantially reduced the transmittance of UV light to PVA@CMC/QL, thus improving the UV barrier performance of PVA@CMC/QL. In addition, the light transmittance of PVA@CMC/QL within the 400- to 800-nm visible light range was substantially improved, facilitating utilization of visible light in photosynthesis and supporting the robust growth of crops beneath the liquid mulch film cover.

Mulch film insulation and moisturizing could increase soil temperature and humidity and promote the growth and development of plants. Liquid mulch film, which is a degradable type of mulch, is primarily employed in arid, cold, and hilly areas for early-stage mulching of crops. By minimizing the evaporation of water and the loss of heat from the soil, liquid mulch film effectively enhances the soil’s moisture retention and insulation properties. The variations in soil moisture and heat at different depths were determined by spraying PVA, PVA@CMC, PVA@QL, and PVA@CMC/QL samples on the soil surface, with a blank sample as a control, respectively (Fig. 4E). At 30 d of observation, the water retention properties of soil at different depths of PVA@CMC/QL were better. The soil moisture corresponding to PVA@CMC/QL ranged from 0.4% to 3.3%, 1.0% to 3.2%, and 0.9% to 3.1%, higher than those of the other control samples at 5-, 10-, and 15-cm soil depths, respectively. This indicated that PVA@CMC/QL prevented the evaporation of water from the soil surface and retained water better than other controls. Meanwhile, the soil insulation performance at different depths of PVA@CMC/QL was better. The soil temperatures corresponding to PVA@CMC/QL ranged from 0.9 to 3.5, 0.4 to 2.4, and 0.4 to 1.9 °C higher than those of the other control samples at 5-, 10-, and 15-cm soil depths, respectively. This is because among all the spray-ability and film-forming tests of liquid mulch films, the integrity and uniformity of the films formed by PVA@CMC/QL were the best. This revealed that the mulch film on the soil surface did stop the soil heat from dissipating to the outside, acting as an effective soil insulator.

### Weed inhibition effect of PVA@CMC/QL

**Fig. 5.**
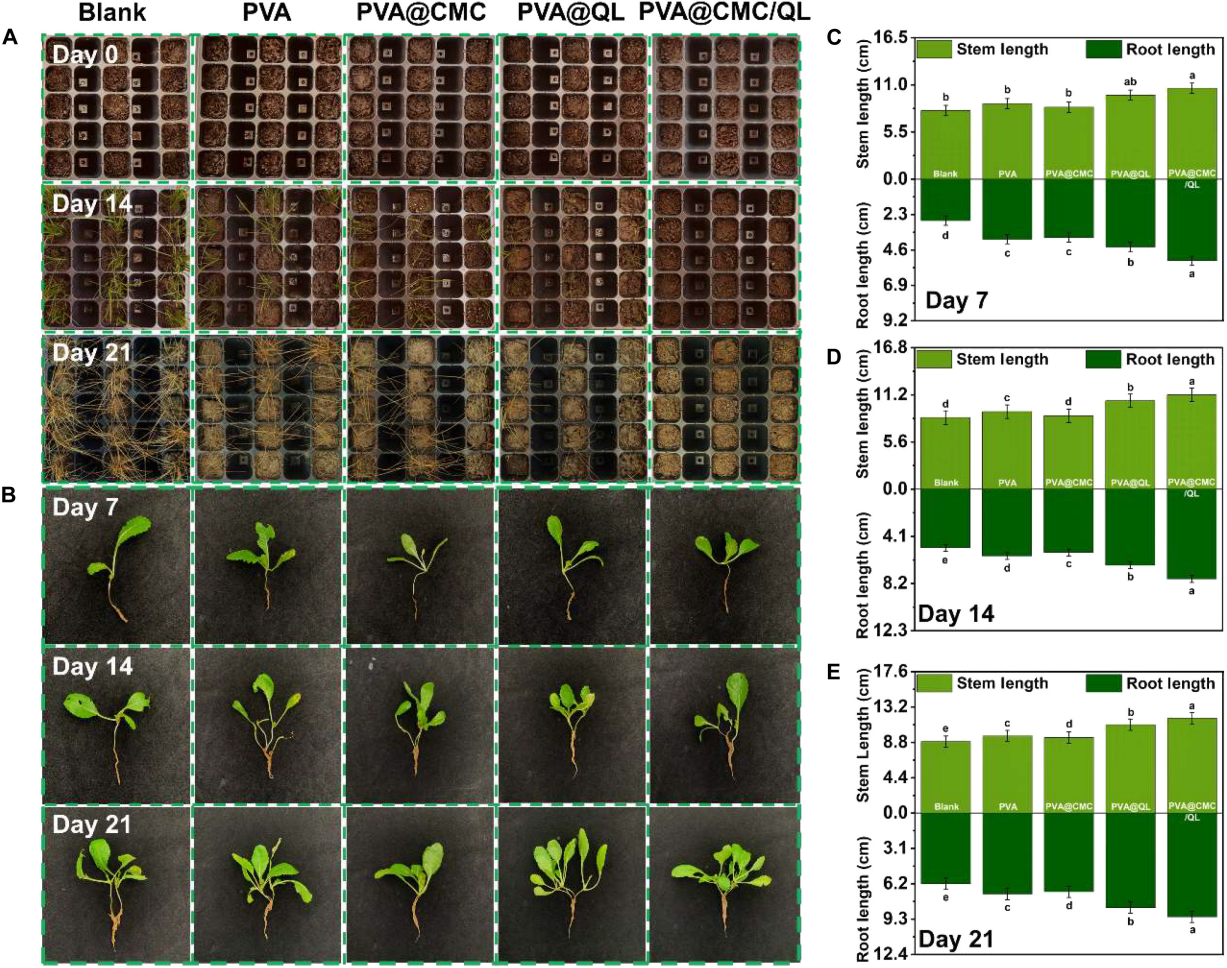
(A) Comparison of the germination rates of weeds in different liquid-mulch-film-covered soils; (B) germination rates of weed seeds in different liquid-mulch-film-covered soils. Changes in the root and stem length of pak choi seedlings in different liquid-mulch-film-covered soils after growth for (C) 7, (D) 14, and (E) 21 d. Statistical analysis was performed using comparison with the control sample. a, b, c, d, and e are used to differentiate statistical significance, with the same letter indicating no difference and different letters indicating a difference. Specific significance of letter labels a to e: Statistical differences between groups showed a trend of decreasing significance from a to e, and groups labeled with the same letter did not show statistically significant differences (*P* > 0.05). Level of significance: *P* < 0.05.

Weeds are highly adaptable, with large root systems, and tend to outcompete crops for essential resources, such as nutrients, water, light, and space, ultimately leading to a decline in both crop output and quality [50]. The effects of 4 types of liquid mulch films on weed emergence and growth inhibition were assessed, with bare soil serving as a comparison group. It is observed in Fig. 5A that by day 14, seeds planted in the holes with bare soil had successfully germinated and grown into seedlings; no germination was observed in the holes covered with PVA@CMC/QL. Some seeds germinated in all 3 groups of PVA, PVA@CMC, and PVA@QL. The weed seedlings in both the bare soil and the other 3 controls were mostly wilted on day 21. A small amount of seedling growth could be seen in the holes covered by PVA@CMC/QL. As mentioned previously, PVA, PVA@CMC, and PVA@QL failed to form into a complete mulch film on the soil, leaving uncovered gaps through which seedlings grew. Weed germination and growth in the PVA@CMC/QL-mulched pots was minimal, and weed growth in the space probably was because regions with a comparatively faint or fragile film layer were formed throughout the remediation process as a result of the unevenness of the soil surface. Weed germination in nursery pots covered with bare soil, PVA, PVA@CMC, PVA@QL, and PVA@CMC/QL was 80.4% ± 2.31%, 49.3% ± 2.31%, 45.7% ± 0.91%, 26.1% ± 1.51%, and 13.3% ± 1.51%, respectively (Fig. S6). Preliminary indications were that several liquid mulch films were able to inhibit weed germination and that the cross-linking robustness of the liquid mulch film along with the extent of soil coverage influenced its ability to inhibit weed growth. Among them, PVA@CMC/QL had the best weed suppression rate.

Pak choi seedlings were sown in soil mixed with weed seeds, and the effects of liquid mulch film protection on crop growth and weed suppression were investigated in a pot experiment. The experimental group was covered with different components of liquid mulch film, with bare soil functioning as a comparison group. The stem length and root length of chard seedlings were measured on a predetermined date to record the growth of the seedlings. As shown in Fig. 5B, the growth conditions of the pak choi seedlings in the experimental group were all better than those in the control group after the 21st day of incubation. The growth condition of the pak choi seedlings was further analyzed by measuring their stem length and root length. The average root lengths of bare soil, PVA, PVA@CMC, PVA@QL, and PVA@CMC/QL chard seedlings were 2.7 ± 0.2, 3.9 ± 0.1, 3.8 ± 0.3, 4.4 ± 0.2, and 5.3 ± 0.3 cm, respectively, on day 7 (Fig. 5C to E). The mean root lengths were 8.0 ± 0.1, 8.8 ± 0.1, 8.4 ± 0.2, 9.8 ± 0.3, and 10.6 ± 0.3 cm. The average stem lengths of bare soil, PVA, PVA@CMC, PVA@QL, and PVA@CMC/QL chard seedlings increased by 3.7 ± 0.1, 3.2 ± 0.1, 3.1 ± 0.2, 3.9 ± 0.3, and 3.8 ± 0.3 cm, respectively, after 21 d of incubation. The mean stem length increased by 0.9 ± 0.1, 0.8 ± 0.1, 1.0 ± 0.2, 1.2 ± 0.3, and 1.2 ± 0.3 cm, respectively. These results further indicated that PVA@CMC/QL-mulched soil significantly inhibited weed growth and promoted pak choi seedling growth. According to the outcomes of the germination of weed seeds and potting tests, along with considerations of manufacturing expenses and ecological safety, PVA@CMC/QL was selected as the best liquid mulch film for further testing.

**Fig. 6.**
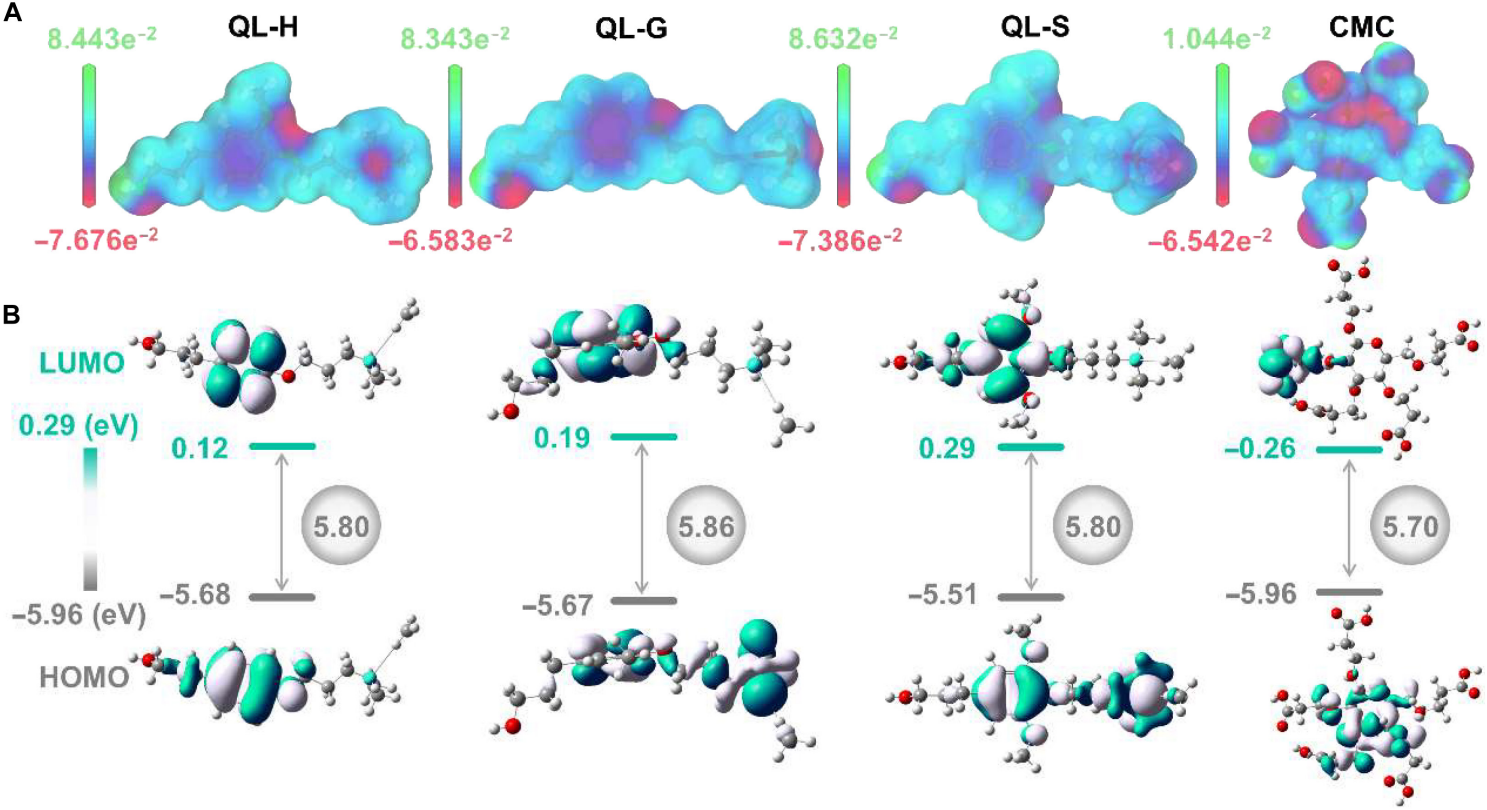
Theoretical calculations for a few compounds modeled on QL and CMC. (A) Molecular surface electrostatic potential (MESP) of *p*-hydroxyphenyl lignin (QL-H), guaiacyl lignin (QL-G), syringyl lignin (QL-S), and CMC model compounds. (B) Molecular frontier orbitals of QL-H, QL-G, QL-S, and CMC model compounds. LUMO, lowest unoccupied molecular orbital; HOMO, highest occupied molecular orbital.

Furthermore, the mechanism of weed suppression by PVA@CMC/QL was explained by analyzing the effect of the inherent structure of biomass molecules on light absorption. The electrostatic potential energy of the QL and CMC model compounds was analyzed based on density functional theory calculations, predominantly impacting on their UV absorption characteristics. Among them, QL calculated 3 structures, namely, *p*-hydroxyphenyl lignin (QL-H), guaiacyl lignin (QL-G), and syringyl lignin (QL-S). Electron density was evaluated by molecular surface electrostatic potential analysis. The predominance of negative molecular surface electrostatic potentials on the exterior of QL and CMC molecules, revealed by their electron-abundant nature, is illustrated in Fig. 6A. The reduction of the energy bandgap (Eg) through the formation of donor–acceptor pairs resulted in a redshift of the absorption spectrum, subsequently contributing to a degradation of UV absorptivity. In biomass polymers, it was discovered that electron-rich monomer condensation produced donor–donor pairs, impeded electron delocalization, and markedly augmented Eg [51]. The results showed that the extent of the redshift in the entire absorption spectrum was reduced, while the ability to absorb UV light was enhanced. Moreover, the highest occupied molecular orbital energy (EHOMO) and the lowest unoccupied molecular orbital energy (ELUMO) of these molecules were examined, and the specific Eg values of several monomers were calculated using the difference between ELUMO and EHOMO. The Eg value (5.86 eV) of QL-G was greater than that of CMC in Fig. 6B, indicating that its UV-absorbing capacity was stronger. It was well explained by these results why QL-added PVA liquid mulch film blocked more UV light than other comparable liquid mulch films, resulting in weed suppression.

### Slow release of selenium fertilizer from PVA@CMC/QL

**Fig. 7.**
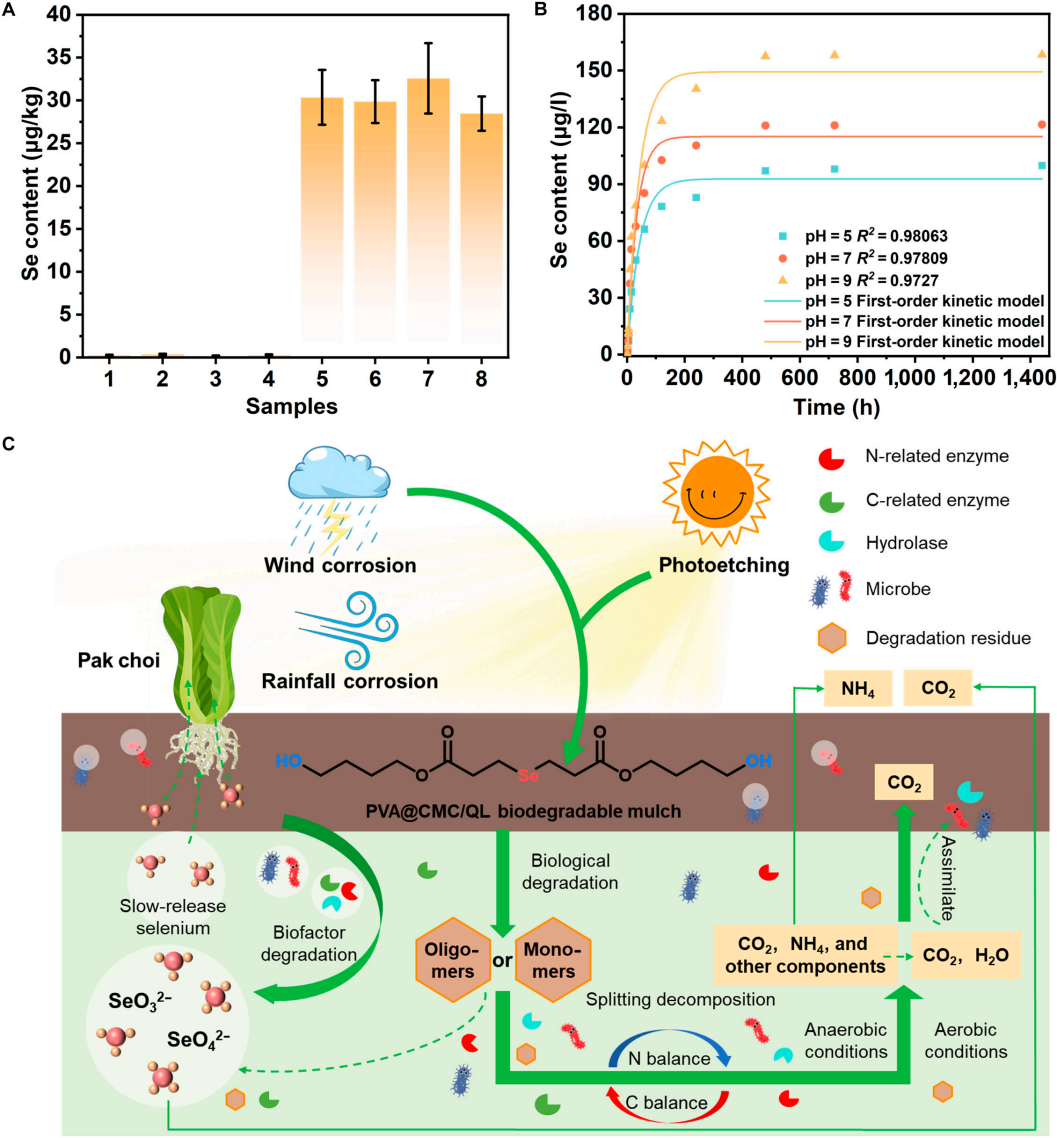
(A) Selenium content in pak choi uncovered (1 to 4) and covered with PVA@CMC/QL (5 to 8). (B) Cumulative release of selenium from PVA@CMC/QL in pH 5.0, 7.0, and 9.0 solutions versus time kinetic curves. The scatter points represent the cumulative release amount, and the curve stands for the first-order kinetic model. (C) Degradation process of PVA@CMC/QL in soil.

The degradation process of mulch film could lead to loss of value, energy consumption, and waste of resources. Liquid slow-release mulch film might be an effective way to prevent loss of value and facilitate waste-to-value conversion. PVA@CMC/QL degradation could serve as a selenium supplement to amend selenium-deficient soils and grow selenium-enriched cash crops, showing significant reutilization value. In order to assess the extent of selenium uptake from degraded PVA@CMC/QL, the selenium content in pak choi was analyzed by inductively coupled plasma emission spectrometry. As illustrated in Fig. 7A, the selenium content of the roots of each sample in the spray group was above 28.5 μg/kg, exceeding the standard for selenium-enriched crops. Compared to the unsprayed group, the sprayed mulch film group had 95 times more selenium. In addition, in order to better understand the selenium-containing slow release of PVA@CMC/QL, the release kinetics of PVA@CMC/QL were tested in aqueous solutions with varying pH values. Figure 7B shows that the selenium-containing slow release of PVA@CMC/QL exhibited the same trend under the 3 different pH conditions. Within the initial 120 min, selenium was released rapidly across all 3 pH conditions, primarily as a result of the rapid dissolution of PVA@CMC/QL. The release gradually slowed down and leveled off after 480 min. Throughout the research period, PVA@CMC/QL demonstrated the greatest selenium release in the pH = 9 solution, while the least amount was released in the pH = 5 solution. The carboxyl group in PVA@CMC/QL existed as COO– under alkaline conditions, and the cross-linked network swelled owing to electrostatic repulsion, facilitating the release of selenium. In contrast, the carboxyl groups of PVA@CMC/QL existed as –COOH under acidic conditions, promoting reinforced hydrogen bond construction in the liquid mulch film configuration and efficiently blocking selenium release. The accumulated release of selenium during the burst release phase at pH = 5, 7, and 9 was 78.2, 102.6, and 123.3 μg/l, respectively, and at the conclusion of the accumulated release experiment, a total of 99.8, 121.4, and 158.3 μg/l of selenium content was released from PVA@CMC/QL. In addition, the higher *R*^2^ (0.98063, 0.97809, and 0.9727) indicated that the selenium release obeyed the kinetic model of diffusion control under different pH conditions.

In addition, PVA@CMC/QL synergistic selenium fertilizer retardation was mainly related to abiotic and biotic factors (Fig. 7C). Abiotic factors included soil temperature and pH, such as degradable mulch film degrading at a higher rate in summer than in winter and oxidative degradation under alkaline conditions. Biological factors were related to microorganisms enriched on the surface of the mulch film, through 3 steps: degradation, cleavage, and mineralization. Biodegradation is defined as a process whereby the physicochemical properties of the membrane are changed by microbial aggregation, UV irradiation, surface oxidation, embrittlement, and so on [52]. Cleavage is the hydrolytic breaking of ester bonds to form oligomers or monomers, which are completely biodegraded to CO_2_ and H_2_O by aerobic microorganisms and converted to small molecules such as CO_2_ and CH_4_ by anaerobic microorganisms. Mineralization is the process by which microorganisms obtain energy and carbon molecules, releasing CO_2_ and H_2_O and producing new biomass. PVA@CMC/QL would release SeO_4_^2−^ and SeO_3_^2−^ after the series of steps described above. Plants had a strong affinity for SeO_4_^2−^ and SeO_3_^2−^, which could be taken up from the soil through the plasma membrane of the root cell and subsequently converted into the organic form of selenium via a series of enzymes in the plant cell.

It was confirmed by these results that PVA@CMC/QL was an effective selenium fertilizer with little crop toxicity. Selenium plays a dual role at different concentrations. Selenium is beneficial to crops and humans at lower concentrations but would inhibit plant growth and exhibit biotoxicity at higher concentrations. By adjusting the amount and level of selenium fertilizer usage, selenium levels in soil or crops might be regulated in actual agricultural production, thereby optimizing advantages while avoiding environmental harm.

### Degradation of PVA@CMC/QL and soil safety assessment

**Fig. 8.**
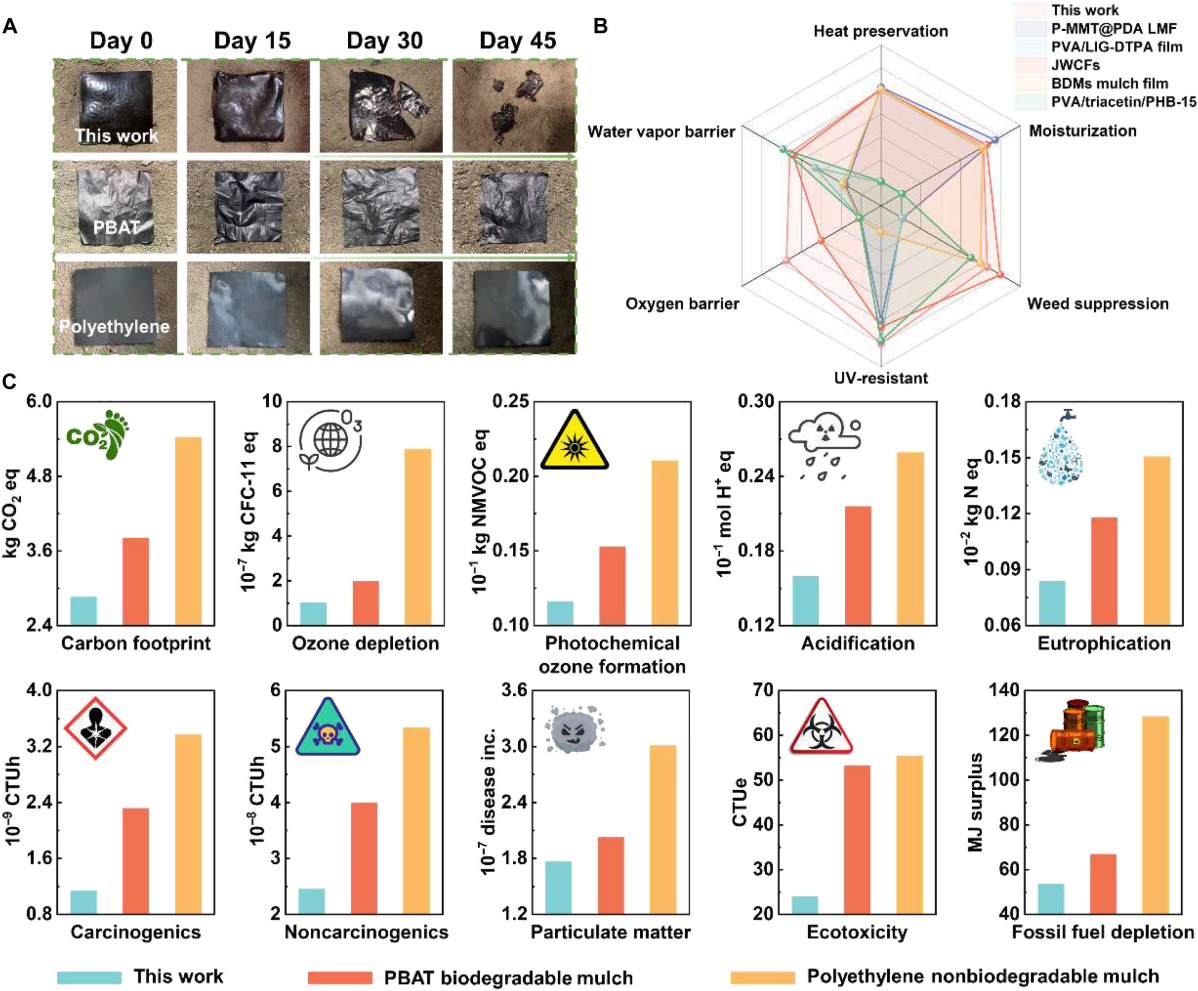
Images of PVA@CMC/QL versus conventional polyethylene and poly(butylene adipate-*co*-terephthalate) (PBAT) mulch films (A) after a 45-d soil burial degradation test. (B) Key properties of PVA@CMC/QL compared to those of other reported mulch films. (C) Life cycle assessment. CTUe, comparative toxic unit for ecosystems; CTUh, comparative toxic unit for human health; CFC-11 eq, equivalent amount of fluorotrichloromethane; NMVOC eq, equivalent amount of non-methane volatile organic compounds.

Degradation performance was an important index for evaluating degradable membranes, with the degradation rate being particularly critical for slow-release membranes [53]. Photographs of PVA@CMC/QL, commercially available biodegradable mulch film (poly(butylene adipate-*co*-terephthalate) [PBAT]), and nonbiodegradable mulch film (PE) after approximately 6 weeks of burial in soil are presented in Fig. 8A. Significant degradation of PVA@CMC/QL occurred around day 30 compared to PBAT and PE. PVA@CMC/QL degradation was up to 84.5% until 6 weeks later (Fig. S7). In contrast, PBAT mulch film had a long degradation cycle with a degradation rate of only 2.2%, and PE mulch film hardly degraded at all. Pak choi could be harvested in about 45 d under the right conditions, as it has a short growth cycle. Slow-release mulch film was time sensitive and needed to be degraded at the right time so that it could be better absorbed and utilized by plants. Compared with other reported mulch films [54–58], PVA@CMC/QL not only had excellent degradability but also had advantages in terms of thermal insulation and moisturization, UV resistance, oxygen barrier, water vapor barrier, and weed suppression (Fig. 8B).

Agricultural mulch film promoted crop yields while ensuring the sustainable use of the soil. This work evaluated the environmental impact of PVA@CMC/QL using LCA and compared it with PBAT and PE (Fig. 8C). The assessment of the environmental performance of PVA@CMC/QL involved the following components: PVA@CMC/QL preparation, extraction of cellulose and lignin from bamboo residues and paper wastes, chemical solvents, water consumption, energy used for film formation, transportation, and end-of-life disposal. The results indicated that the upstream production of chemicals used in the preparation of PVA@CMC/QL was the main contributor to the impact category, for example, fossil fuels, biotoxicity, and the ozone layer. PVA@CMC/QL had lower environmental impacts than PBAT and PE in all impact categories.

## Conclusion

In this research, a biodegradable bamboo-based liquid slow-release mulch film was prepared from bamboo residues. The obtained PVA@CMC/QL could be simply sprayed upon the outer layer of the soil to construct a solid mulch film, which offered excellent moisture retention, heat insulation, oxygen barrier properties, UV resistance, and weed suppression. These attributes provided significant support for creating an optimal growth environment for the pak choi. It also elucidated the ability of PVA@CMC/QL to absorb UV in its molecular structure, thereby achieving weed suppression, as demonstrated by density functional theory. In addition, PVA@CMC/QL had remarkable selenium fertilizer slow-release capability, which could be used for various potential applications such as selenium-enriched vegetables and improvement of selenium-deficient soil. Finally, LCA analysis showed that PVA@CMC/QL was more environmentally friendly than commercially available PBAT and PE. As a multifunctional, biodegradable liquid slow-release mulch film, PVA@CMC/QL offers optimized conditions for crop growth by enhancing moisture retention, thermal insulation, weed suppression, and the controlled release of selenium fertilizer. In addition to promoting the high-value utilization of bamboo residues, PVA@CMC/QL addresses the issue of the limited functionality of conventional biodegradable mulch films. With its promising potential as an ideal alternative to traditional plastic mulch film, this innovative material holds significant advantages for environmental sustainability and carbon footprint reduction.

## Materials and Methods

### Materials

Bamboo powder was obtained from Changsha Yuantian Bamboo Products Co. Kraft lignin hydrochloride was purchased from Hubei Shuaiyan Ligao Biomedical Co. PVA (type 1788), (3-chloro-2-hydroxypropyl) trimethyl ammonium chloride (CHPTMAC), and 1,4-butanediol were obtained from Aladdin Reagent. Sodium periodate, sodium chloroacetate, 3-chloropropionyl chloride, and selenium powder were purchased from Macklin Reagent. Sodium borohydride, NaOH, sulfuric acid, tetrahydrofuran, and acetic acid were purchased from Sinopharm Reagent Co. All reagents did not require any additional purification process.

### Preparation of PVA@CMC/QL

Cellulose extracted from bamboo powder was prepared by sulfuric acid hydrolysis to obtain nanocellulose; 5.0 g of nanocellulose and 80 ml of anhydrous ethanol were taken, and 20 ml of 15 wt% aqueous NaOH solution was added, followed by stirring for 60 min at 30 °C in a water bath. Then, 5.0 g of sodium chloroacetate was added, the heat was raised to 65 °C, and the reaction proceeded for a duration of 2.5 h. After the reaction was completed, the pH of the solution was modified using acetic acid, bringing it to a range of 7 to 8. Subsequently, the solution was pump-filtered, and the solid was washed with ethanol several times, dried under vacuum at 65 °C for 18 h, and pulverized to obtain CMC samples; 5.0 g of kraft lignin was dissolved in 20 ml of 20 wt% aqueous NaOH, and 9 g of CHPTMAC was added slowly dropwise under continuous stirring. Following the addition, the temperature of the mixture was increased to 85 °C and then maintained for 4 h. Aqueous NaOH solution was added dropwise to keep the reaction under alkaline conditions during the reaction. The solution was dialyzed and freeze-dried to obtain a QL sample after the reaction was completed. The synthesis of bis(4-hydroxybutyl)-3,3′-selenodipropionate (SeC_2_CO) is documented in the Supplementary Materials (Fig. S1).

Subsequently, 5 wt% CMC and 15 wt% QL were taken and dissolved in an aqueous solution and stirred vigorously for 2 h at room temperature to obtain a mixed CMC/QL solution. CMC/QL was mixed with an equal amount of PVA (5 wt%) solution, and the reaction was conducted at 80 °C for a duration of 4 h, with the addition of 4 wt% SeC_2_CO. PVA@CMC/QL samples were collected after the reaction was completed, and the temperature was allowed to return to room temperature. In addition, the PVA@CMC/QL solution was poured into a polytetrafluoroethylene mold and naturally air-dried to form a film at room temperature for subsequent experimental examination.

### Characterization

A scanning electron microscope (TESCAN MIRA4) with an energy spectrometer was used to image the morphology of the different samples and to analyze the elemental distribution on their surfaces. XPS (Thermo K-Alpha) was employed to analyze the chemical composition of the thin film samples. Response surface optimization was conducted using a central composite design model with 2 factors and 3 levels, which included 5 central points and resulted in 13 experimental runs. The surface functional groups of the samples were characterized using a Fourier transform infrared spectrometer (Nicolet iS 10), which operated with a spectral resolution of 2 cm^−1^ and a measurement range of 4,000 to 500 cm^−1^. Operated at a scan rate of 1°/min and a 2*θ* range of 5° to 90°, an X-ray diffractometer (X’Pert PRO MPD) was used to perform x-ray diffraction research. In addition, a thermogravimetric analyzer (Netzsch TG 209 F1) was used to research the thermal stability of different samples. In the thermogravimetry analysis/derivative thermogravimetry testing, the N_2_ flow rate was set to 20 ml/min, with a temperature range of 25 to 600 °C and a heating rate of 10 °C/min. The permeability of PVA@CMC/QL was characterized using a Labthink BTY-B2P permeability tester. A contact angle meter was also utilized to test the water droplet contact angle of PVA@CMC/QL following its film formation on the soil surface.

### Mechanical properties

In accordance with the GB/T 1040 standard, a CMT6103 electronic universal testing machine was employed to measure the tensile strength and elongation at break of PVA@CMC/QL. Furthermore, Young’s modulus was derived via fitting the slope of the initial linear portion of the stress–strain curve. The mulch films were prepared with a width of 10 mm, a length of 50 mm, and a thickness of 0.5 mm. After being dehydrated to a constant mass at 80 °C, the mulch films were clamped in a tester fixture and stretched at indoor temperature, at a strain rate of 20 mm/min. Each sample underwent 4 iterations of the test, and the average value was used to determine the outcome [59].

### Viscosity, wicking, sprayability, and film formation tests

The viscosity of all liquid mulch films was evaluated using a rheometer (HAAKE MARS 60) at speeds from 0 to 1,000 rpm at 25 °C. A 15-mm-diameter, 50-mm-high glass jar was filled with 20 g of 2-mm-sieved soil. Then, the soil surface was moistened with 1.5 ml of water to reduce its hydrophobicity and allow the liquid mulch film to coat the top layer of the soil. A total of 2 ml of PVA@CMC/QL was added drop by drop to the soil surface, and the depth of penetration of the liquid mulch film was monitored [60].

The spray performance and film formation characteristics of PVA@CMC/QL were tested by wetting on the top layer of the soil in a glass petri dish with a limited quantity of water and spraying a liquid mulch film onto the soil surface via a handheld spray gun. In addition, the amount of liquid mulch film sprayed was 0.92 kg/m^2^.

### UV barrier properties

PVA@CMC/QL’s light barrier characteristics were tested using a UV–visible spectrophotometer. Rectangular samples (30 × 30 mm) were constructed to measure absorbance between 300 and 900 nm [61]. The light barrier qualities were examined by transmittance (UV and visible) and opacity, which were computed using Eq. 1:

$$\text{Opacity}\;\left({\mathrm{mm}}^{-1}\right)=\frac{\mathrm{AU}}{d}$$ (1)

where *AU* denotes the absorbance of the mulch film at distinct light wavelengths; *d* (mm) denotes the thickness of the mulch film.

### Soil moisturizing and heat preservation tests

Before the experiments, the soil in potted plants was loosened and leveled, and PVA@CMC/QL was sprayed homogeneously onto the soil surface to form mulch films. A soil temperature and moisture tester was utilized to test soil moisture as well as the thermal condition at 5-, 10-, and 15-cm depths. Data results were averaged using a 3-point sampling method with multiple measurements. The experiments were conducted every 5 d from 1400 to 1500 [62].

### Weed growth inhibition tests

The inhibitory effect of different compositions of PVA@CMC/QL on weed germination was investigated. The seed trays consisted of a 5 × 5 hole configuration, with each hole measuring 50 × 50 mm^2^ in area. Ten of the 25 holes were left empty to allow for additional observation, while 15 holes from columns 1, 3, and 5 were planted with approximately 30 weed seeds per hole. Images were taken on days 0, 14, and 21 to track the germination of seeds every day following the application of liquid mulch film [63]. Eq. 2 was used to determine the weed germination ratio of each unique PVA@CMC/QL during the triplicate experiment:

$$\text{Weed germination rate}\;\left(\%\right)=\frac{W_{1}}{W_{0}}\times 100\%$$ (2)

where *W*_0_ is the total number of seeded holes, which in this instance was 15, and *W*_1_ is the number of holes where weeds grew on a given day.

### Selenium fertilizer slow-release tests

PVA@CMC/QL was sprayed during the seedling period of pak choi, and samples were taken at 15-d intervals. Inductively coupled plasma emission spectrometry was employed to further analyze the selenium content of the pak choi.

### Biodegradation

PVA@CMC/QL, along with commercially available biodegradable and nonbiodegradable mulch films, was additionally placed at a 10-cm depth in the experimental plots. In this case, the temperature range was from 15 to 28 °C and the humidity range was from 50% to 70% of the water held in the field. Samples were retrieved every 15 d, followed by washing their surfaces with deionized water. This process guaranteed that all visible soil particles were removed [64]. Subsequently, the samples were subjected to desiccation at 55 °C until they reached a constant weight, after which the biodegradation rate was determined using Eq. 3; the result was indicated as

$$\text{Degradation rate}\;\left(\%\right)=\frac{D_{0}-D_{1}}{D_{0}}\times 100\%$$ (3)

where *D*_0_ (g) represents the mulch film’s original mass and *D*_1_ (g) represents the dry mulch film’s weight at various points in time.

## Data Availability

Data will be made available on request.

## Supplementary Materials

Supplementary 1 Graphical Abstract Figs. S1 to S8 Table S1
