# Supplementary material for: Biodegradable Liquid Slow-Release Mulch Film Based on Bamboo Residue for Selenium-Enriched Crop Cultivation
Source: Research (Wash D C). 2025 May 12;8:0685. doi: 10.34133/research.0685 (PMC12067929; doi:10.34133/research.0685)
Supplement: Supplementary 1 — Graphical Abstract Figs. S1 to S8 Table S1 [file research.0685.f1.zip › research.0685.f1.docx]

**Supplementary Materials**

**Biodegradable liquid slow-release mulch film based on bamboo residue for selenium-enriched crop cultivation**

Chaoqi Chen ^a^, Zhaoshuang Li ^a, *^, Kuaile Yin ^a^, Lei Li ^a^, Zhen Zhang ^a^,Xu Xu ^b^, He Liu ^c^, Yan Qing ^a^, Xingong Li ^a^, Yiqiang Wu ^a, *^

*^a^ College of Materials Science and Engineering, Central South University of Forestry & Technology, Changsha 410004, China*

*^b^ International Innovation Center for Forest Chemicals and Materials, Nanjing Forestry University, Nanjing 210037, China*

*^c^ Institute of Chemical Industry of Forestry Products, Chinese Academy of Forestry, Key Laboratory of Biomass Energy and Material, Nanjing 210042, Jiangsu Province, China*

* Corresponding author: Zhaoshuang Li ([lzs0829@csuft.edu.cn](mailto:lzs0829@csuft.edu.cn)) & Yiqiang Wu ([wuyq0506@126.com](mailto:wuyq0506@126.com))


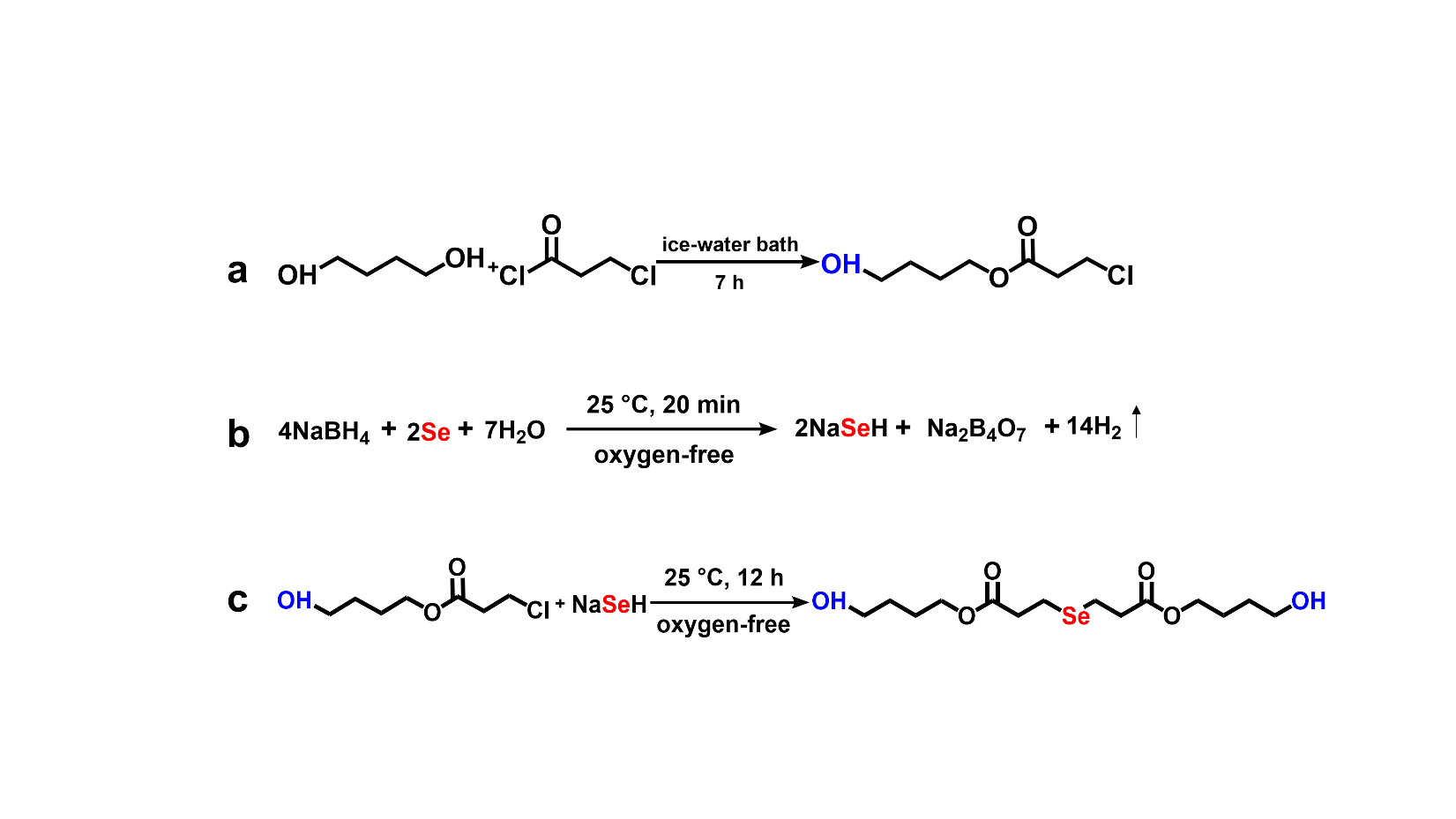


**Fig. S1.** Synthesis of bis(4-hydroxybutyl)-3,3'-selenodipropionate (SeC_2_CO).

The synthesis of SeC_2_CO was referenced from previous studies by He et al [45]. and Zhang et al [41]. As shown in Fig. S1a, 29.64 mmol of 1,4-butanediol was taken and added to 20 mL of tetrahydrofuran solution and stirred under ice water bath conditions. Subsequently, 29.64 mmol 3-chloropropionyl chloride was added slowly and dropwise to the above liquid. The solution was stirred at room temperature for 7 hours. The 4-hydroxybutyl-3chloropropionate (HOC_2_Cl) product was purified by column chromatography.

As in Fig. S1b, sodium borohydride (2.34 g) and selenium powder (2.24 g) were dissolved in 50 mL of deionized water and the reaction was carried out under nitrogen atmosphere. The reaction mixture was stirred at room temperature and the black selenium powder slowly changed to a colorless solution within 20 min (NaHSe).

The HOC_2_Cl solution was injected into the NaHSe solution as shown in Fig. S1c. The mixture was stirred at room temperature for 12 h under N_2_ atmosphere. The product was purified by column chromatography to give the yellow liquid SeC_2_CO.


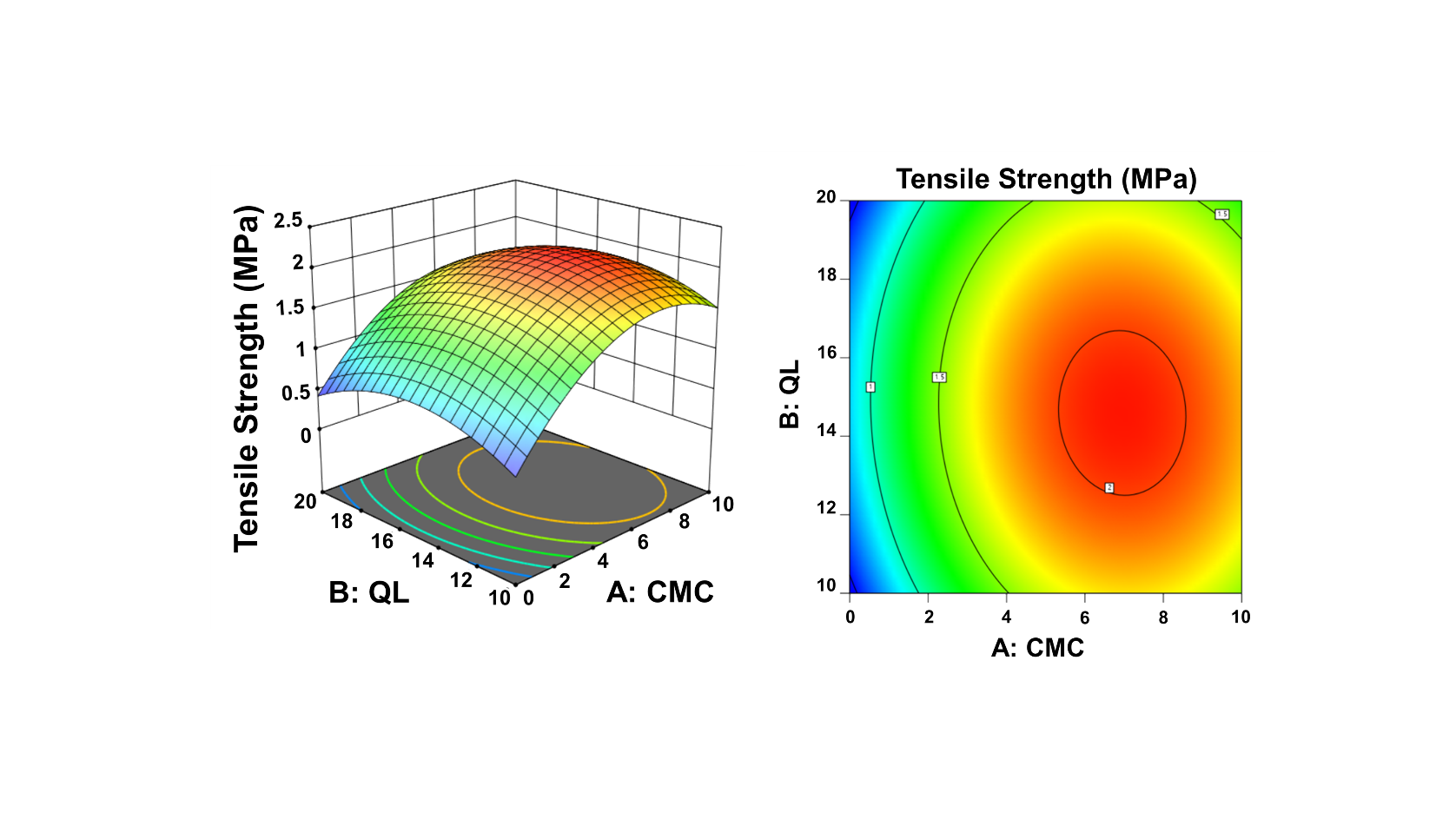


**Fig. S2.** Optimal ratio of CMC to QL in PVA@CMC/QL optimized by response surface methodology.


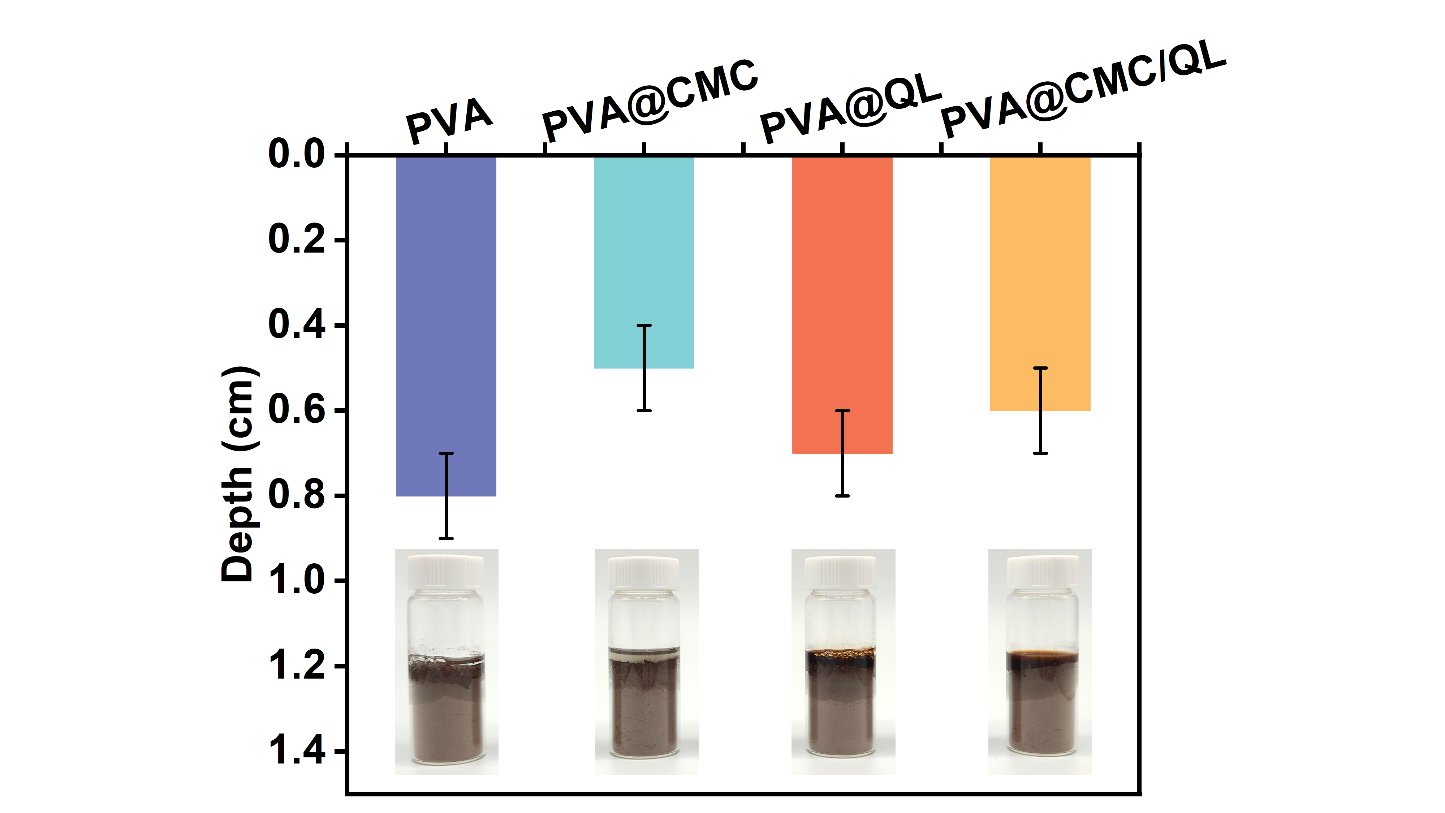


**Fig. S3.** Penetration depth of different samples under soil in wicking tests.


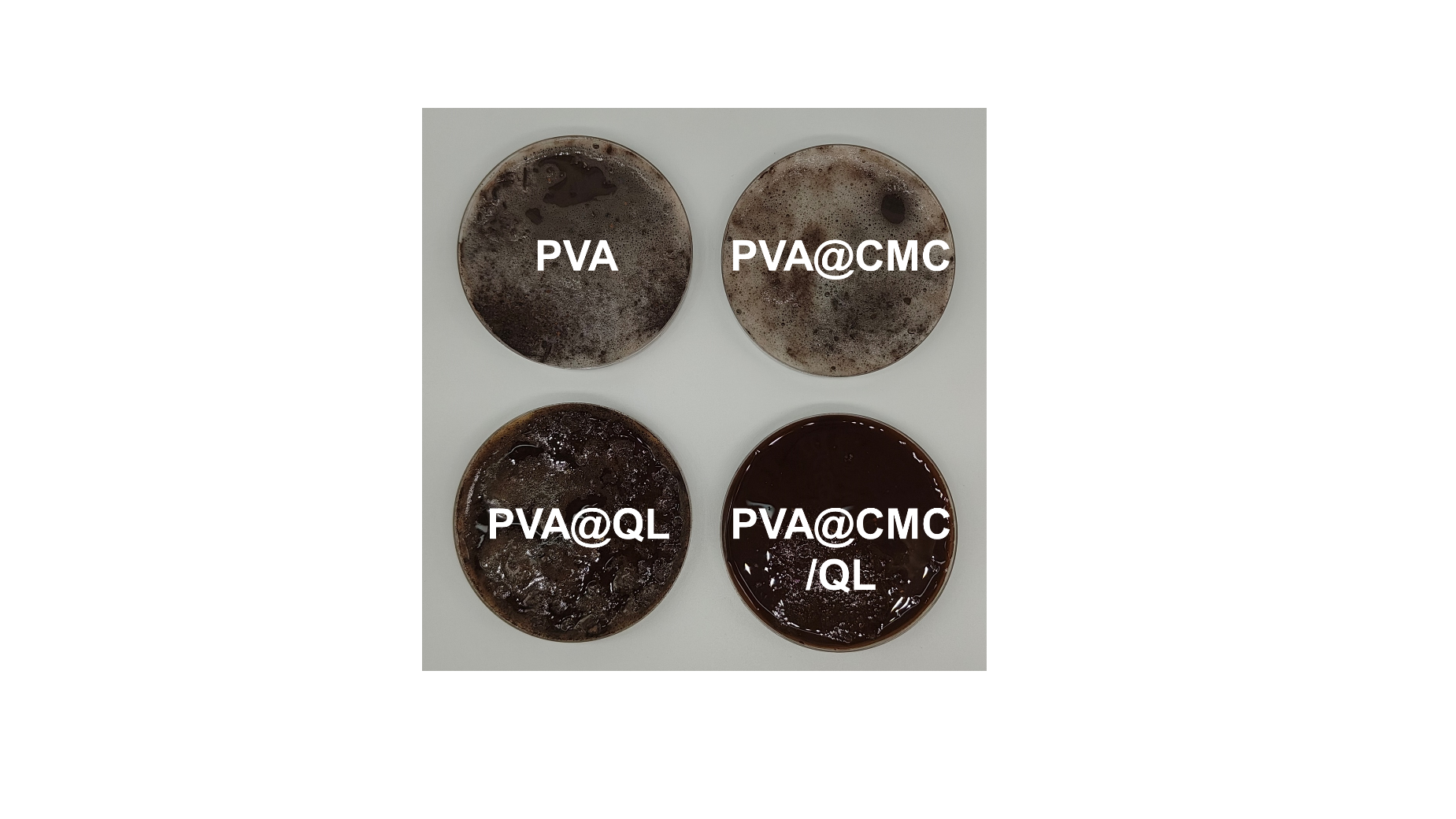


**Fig. S4.** Films formed by different samples on the soil surface of the petri dishes.


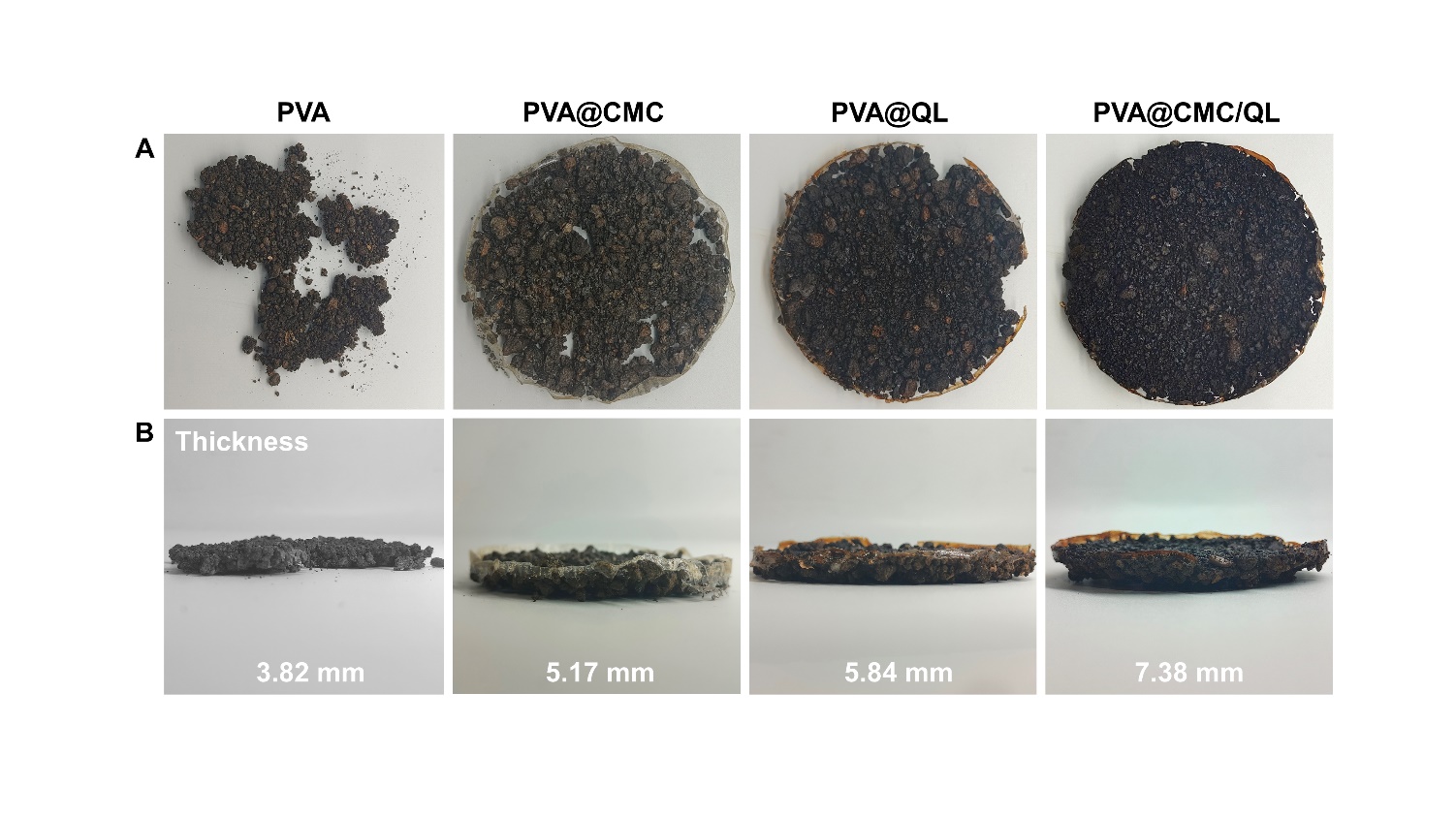


**Fig. S5.** Different liquid mulch films form clods on the soil surface. (A) Overhead image with (B) thickness.


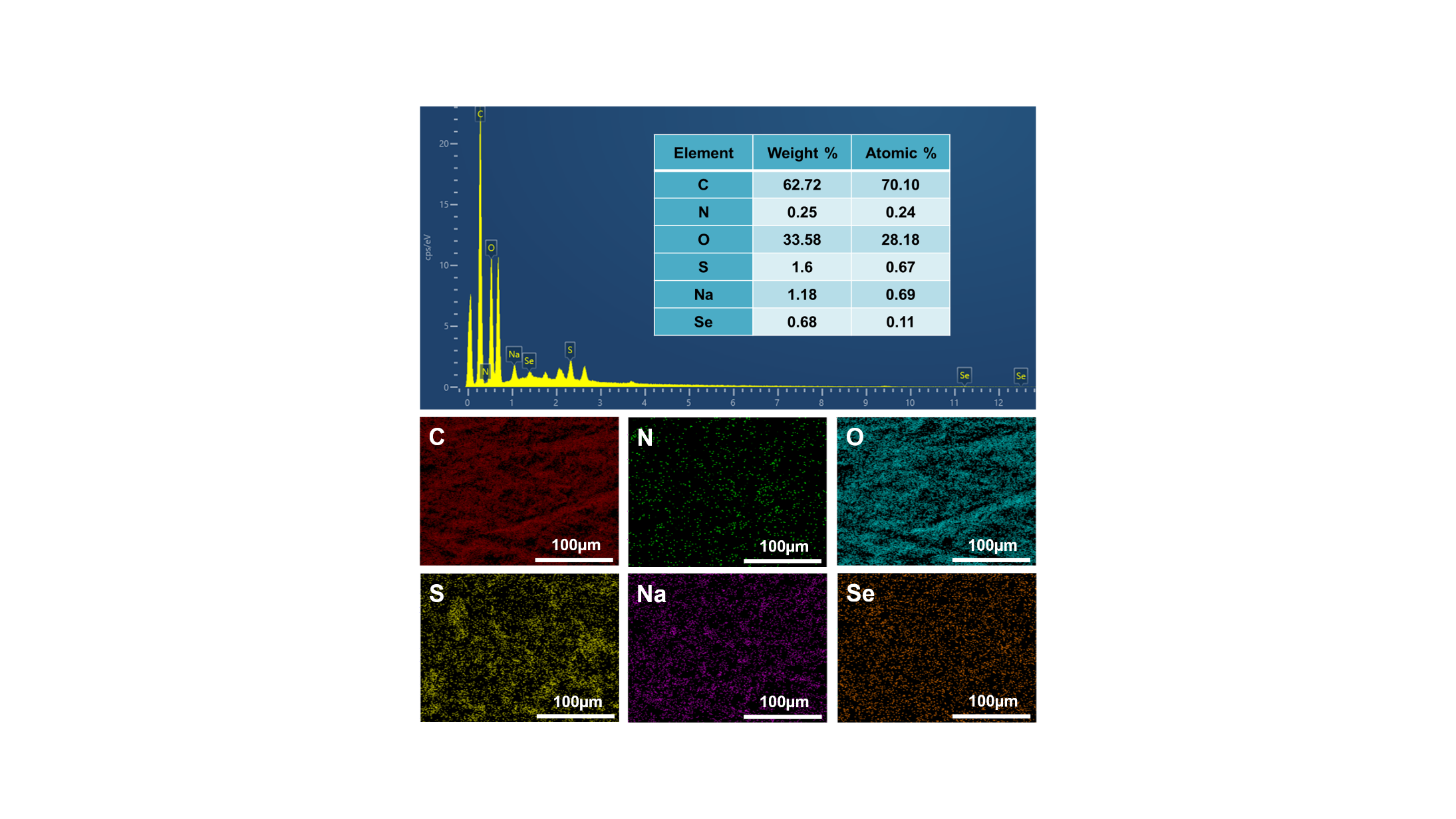


**Fig. S6.** EDS patterns of PVA@CMC/QL.

**Table S1.** TGA information of different samples under nitrogen atmosphere.

| Samples | T_5%_ (℃) | T_max_ (℃) | R_max_ (%/min) | Residue at 600℃ (%) |
| --- | --- | --- | --- | --- |
| PVA | 293.2 | 328.4 | 8.44 | 10.15 |
| PVA@CMC | 276.8 | 305.1 | 5.79 | 27.62 |
| PVA@QL | 248.3 | 281.7 | 3.63 | 27.47 |
| PVA@CMC/QL | 232.5 | 282.9 | 5.21 | 32.78 |


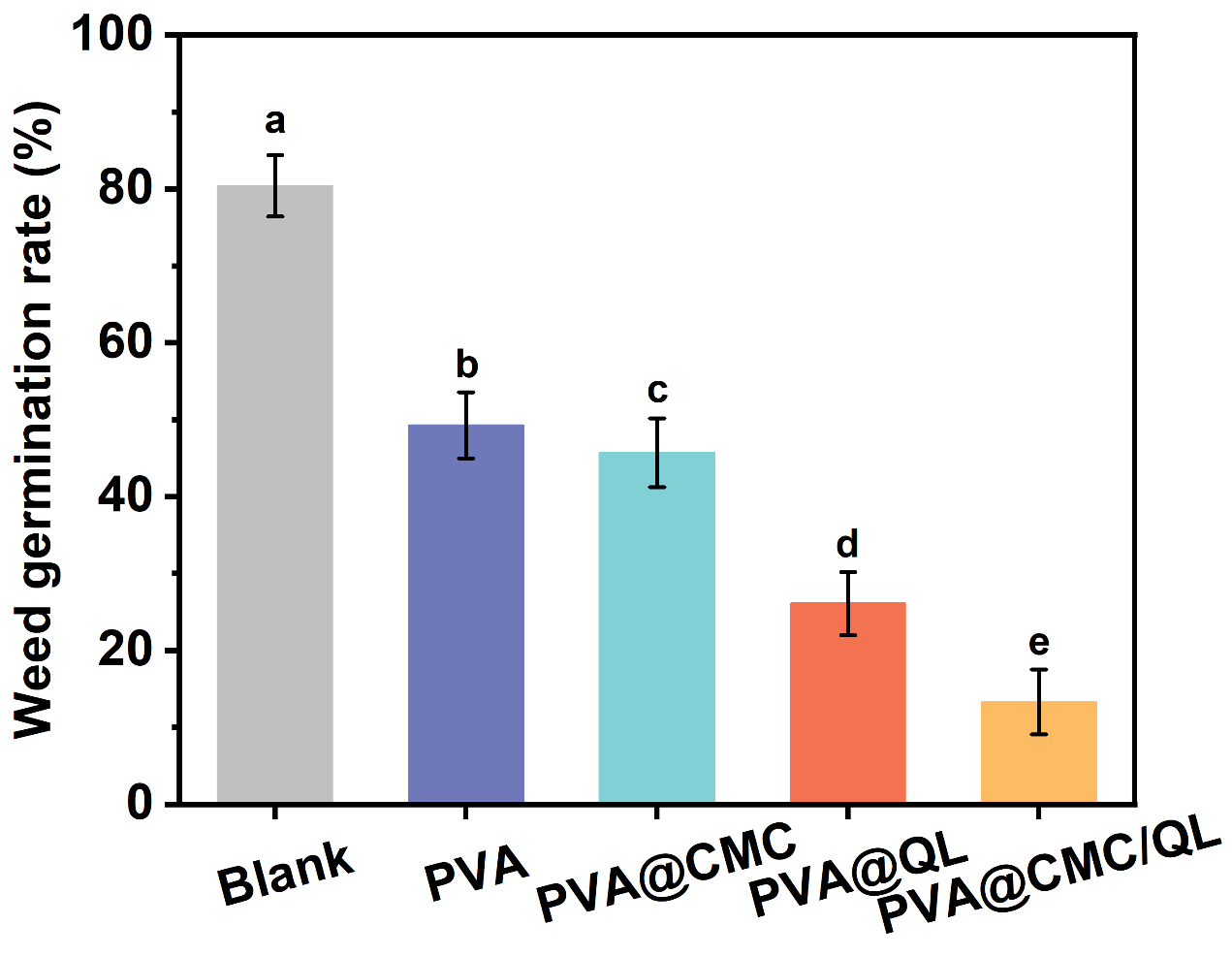


**Fig. S7.** Germination of weed seeds in different liquid-covered soils. Statistical analysis was performed using comparison with the control sample. Levels of significance: p < 0.05.


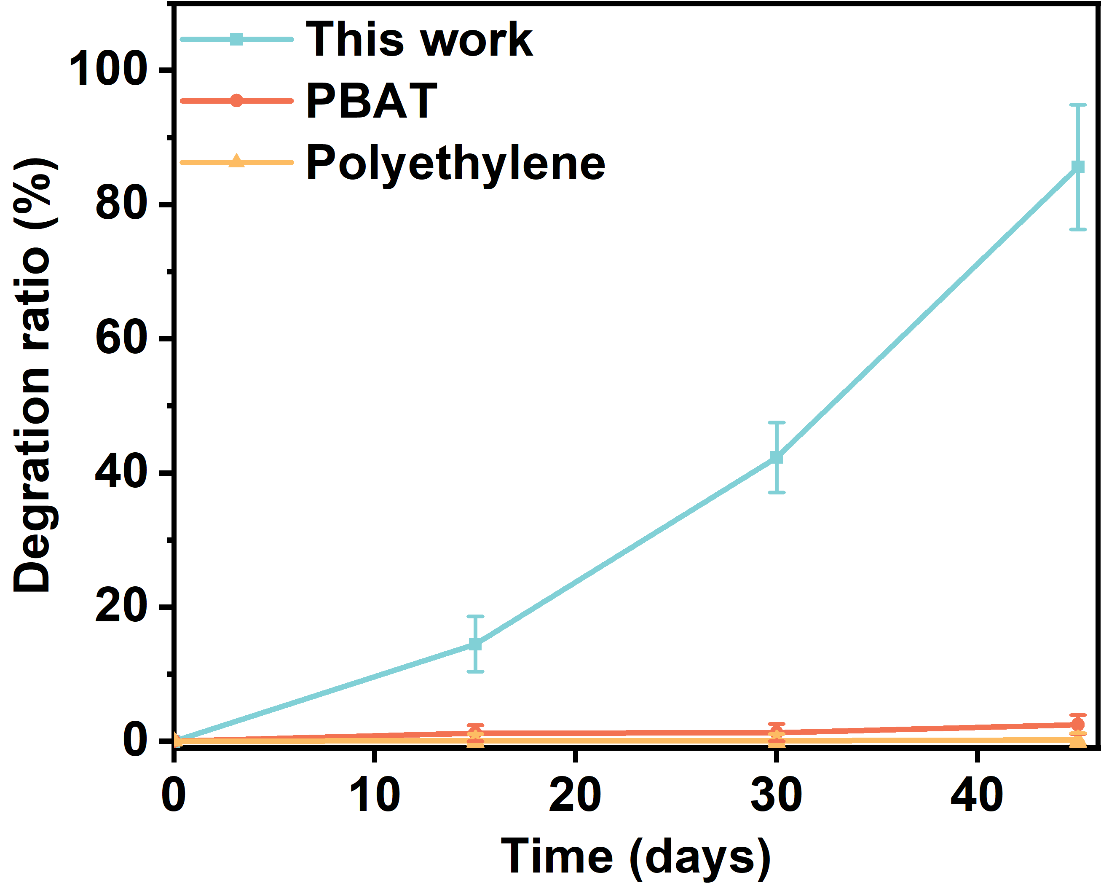


**Fig. S8.** Degradation rate of PVA@CMC/QL vs. conventional PE, PBAT mulch after 45 days soil burial experiment.

***Reference***

41. Zhang XW, Cheng FY, Guo JW, Zheng SM, Wang X, Li SY. Enzymatic synthesis of organoselenium compounds via C‒Se bond formation mediated by sulfur carrier proteins. *Nat. Synth.* 2024;3(4):477-487.

45. He CW, Liu C, Pan SJ, Tan YZ, Guan J, Xu HP. Polyurethane with β‐Selenocarbonyl Structure Enabling the Combination of Plastic Degradation and Waste Upcycling. *Angew. Chem. Int. Ed.* 2024;63(7):e202317558.
