# Supplementary figures and images for: Biodegradable Liquid Slow-Release Mulch Film Based on Bamboo Residue for Selenium-Enriched Crop Cultivation
Source: Research (Wash D C). 2025 May 12;8:0685. doi: 10.34133/research.0685 (PMC12067929; doi:10.34133/research.0685)

**Graphical abstract**

**
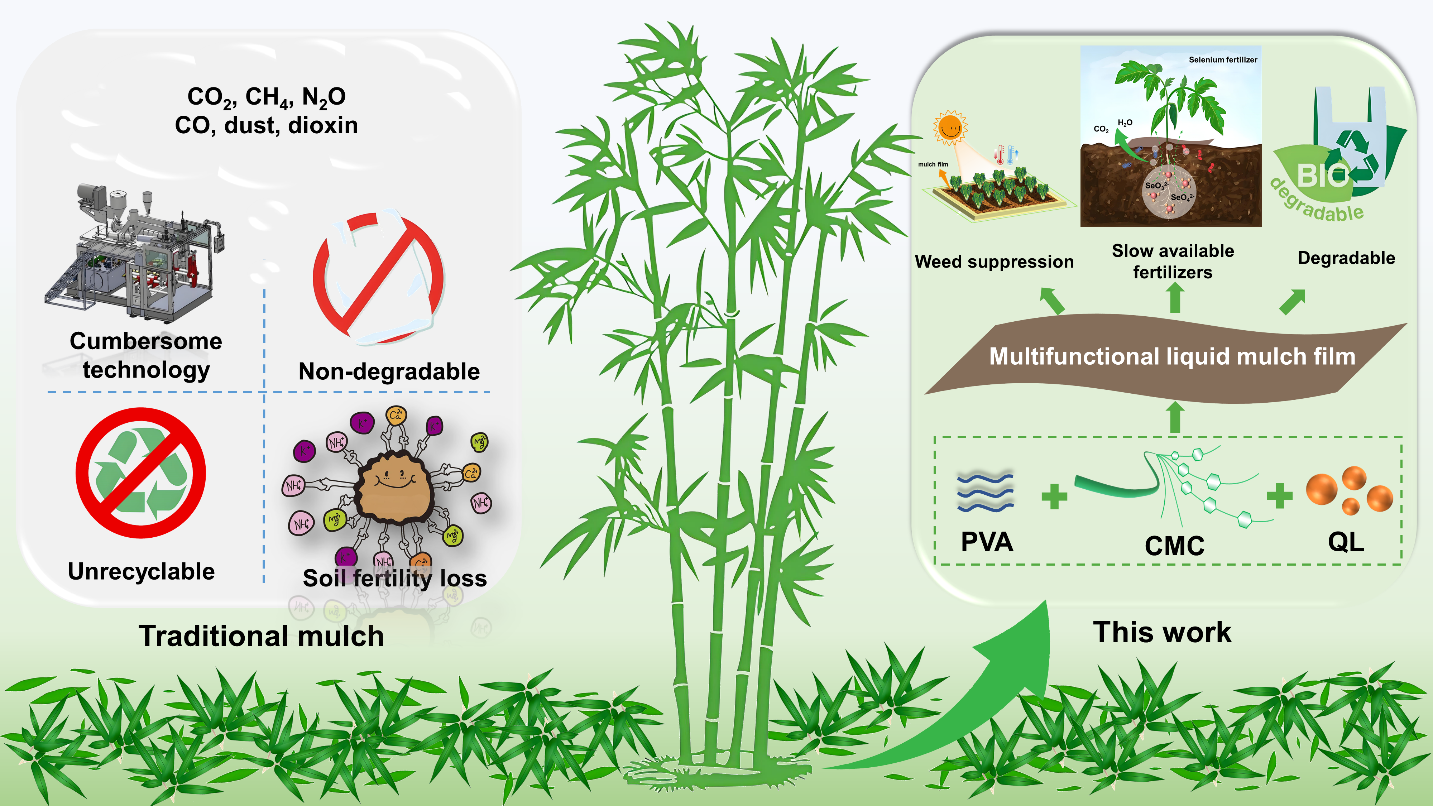
**

Supplement: Supplementary 1 — Graphical Abstract Figs. S1 to S8 Table S1 [file research.0685.f1.zip › Graphical abstract.docx]
